# Plant genotypic diversity and defoliation jointly shape root exudation, fungal communities, and carbon and nitrogen cycling

**DOI:** 10.1111/nph.71479

**Published:** 2026-08-02

**Authors:** Irene Cordero, Mathilde Chomel, Ellen L. Fry, Agnès Ardanuy, Ully Kritzler, Hayley Craig, Cristina Heredia‐Acuña, Sarah Worthington, Stênio I. A. Foerster, Richard D. Bardgett, David Johnson, Marina Semchenko

**Affiliations:** ^1^ Department of Earth and Environmental Sciences The University of Manchester Oxford Road Manchester M13 9PT UK; ^2^ Swiss Federal Institute for Forest, Snow and Landscape Research WSL Zürcherstrasse 111 Birmensdorf CH‐8903 Switzerland; ^3^ FiBL France, Research Institute of Organic Agriculture Eurre 26400 France; ^4^ Univ Toulouse, CNRS, INRAE LIPME 24 Chemin de Borde Rouge, Auzeville, 31326 Castanet Tolosan cedex Toulouse 52627 CS France; ^5^ Lancaster Environment Centre Lancaster University Bailrigg LA1 4YQ UK; ^6^ Department of Education Education Shared Business Services (ESBS) Centre Floor 6, The Generali Building, Blanchardstown Retail Park, Blanchardstown Dublin 15 D15 YT2H Ireland; ^7^ Institute of Ecology and Earth Sciences University of Tartu J. Liivi 2 Tartu 50409 Estonia; ^8^ Department of Ecology Swedish University of Agricultural Sciences Ulls väg 16 Uppsala 756 51 Sweden

**Keywords:** *Anthoxanthum odoratum*, carbon cycling, defoliation, fungal pathogens, grazing, nitrogen cycling, plant genetic diversity, root exudation

## Abstract

- Intraspecific plant diversity is a major component of community diversity, yet its role in shaping plant–microbial interactions and soil functioning remains poorly understood. In particular, plant genotypic diversity may modulate ecosystem responses to defoliation, a key driver of ecosystem processes in grazed systems.
- We subjected genotypic monocultures and mixtures of the grass *Anthoxanthum odoratum* to defoliation and quantified carbon fluxes, root exudation, and soil enzymatic activity. Using stable isotope labelling, we traced the movement of litter‐derived ^15^N and photosynthetically derived ^13^C between plants and soil. The composition of soil fungal communities was assessed using ITS metabarcoding.
- Both defoliation and genotypic diversity increased root exudation, which was positively correlated with soil enzymatic activities and plant uptake of litter‐derived ^15^N. The relative benefit of enhanced N mineralisation under defoliation depended on plant genotypic diversity: incorporation of ^15^N into leaves relative to ^15^N incorporation into soil microbial biomass was significantly higher in genotypic mixtures than in monocultures. Higher plant genotypic diversity was associated with lower soil fungal diversity, while defoliation increased saprotroph abundance.
- Plant genotypic diversity and defoliation jointly regulate plant–microbial interactions, with genotypic diversity enhancing plant performance under defoliation by shifting the partitioning of mineralised N from soil microbial biomass to plants.

## Graphical abstract

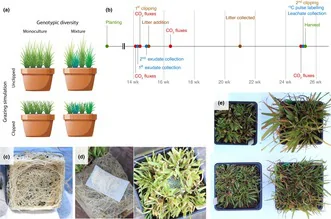

## Resumen

- La diversidad intraespecífica de las plantas es un componente fundamental de la biodiversidad; sin embargo, su papel en la configuración de las interacciones entre plantas y microorganismos y en el funcionamiento del suelo sigue sin conocerse bien. En particular, la diversidad genotípica de las plantas puede modular las respuestas del ecosistema a la defoliación, un factor clave que impulsa los procesos ecosistémicos en pastizales.
- Monocultivos genotípicos y mezclas de la gramínea *Anthoxanthum odoratum* fueron sometidos a defoliación y cuantificamos los flujos de carbono, la exudación radicular y la actividad enzimática del suelo. Mediante el marcaje con isótopos estables, seguimos el movimiento del ^15^N procedente de hojarasca y del ^13^C de origen fotosintético hacia las plantas y el suelo. La composición de las comunidades fúngicas del suelo se evaluó mediante metabarcoding del ITS.
- Tanto la defoliación como la diversidad genotípica aumentaron la exudación radicular, que se correlacionó positivamente con las actividades enzimáticas del suelo y la absorción por parte de las plantas del ^15^N procedente de la hojarasca. El beneficio relativo de una mayor mineralización del nitrógeno bajo condiciones de defoliación dependió de la diversidad genotípica de las plantas: la incorporación de ^15^N en las hojas, en relación con la incorporación de ^15^N en la biomasa microbiana del suelo, fue significativamente mayor en las mezclas genotípicas que en los monocultivos. Una mayor diversidad genotípica de las plantas se asoció con una menor diversidad fúngica del suelo, mientras que la defoliación aumentó la abundancia de hongos saprótrofos.
- La diversidad genotípica de las plantas y la defoliación regulan conjuntamente las interacciones entre plantas y microorganismos, y la diversidad genotípica mejora el rendimiento de las plantas en condiciones de defoliación al desplazar la distribución del nitrógeno mineralizado de la biomasa microbiana del suelo hacia las plantas.

## Introduction

Intraspecific plant diversity is a major but often overlooked component of biodiversity (Westerband *et al*., 2021). Intraspecific variation in plant height and leaf and root morphological traits can be comparable in magnitude to interspecific variability within communities (Herz *et al*., 2017; Buchmann *et al*., 2018; Roybal & Butterfield, 2019) and can be a key factor explaining grassland productivity (Zhang *et al*., 2022) and plant community responses to global change (Henn *et al*., 2018; Yan *et al*., 2026). However, how intraspecific diversity shapes the response of ecosystems to biotic perturbations, such as defoliation by grazing, is still unclear. In grassland ecosystems, grazing by large herbivores is the main biotic factor that shapes vegetation dynamics and ecosystem processes above and below ground (Milchunas & Lauenroth, 1993; Kaarlejärvi *et al*., 2017; Wang *et al*., 2019), with plant responses to defoliation being a key component of grazing effects on ecosystems (Bardgett & Wardle, 2003). Plants can enhance their nitrogen (N) uptake and effectively regrow leaves following a defoliation event by stimulating soil microbial activity and N mineralisation from organic matter through increasing root exudation, which provides labile carbon to microbial decomposers (Hamilton III & Frank, 2001; Sun *et al*., 2017). However, responses to defoliation are highly variable and context‐dependent. While interspecific variation in responses to defoliation is well documented (Mikola *et al*., 2001; Medina‐Roldán & Bardgett, 2011; Liu *et al*., 2023), the role of intraspecific diversity among interacting plants in modulating plant and soil responses to defoliation is still poorly understood despite its importance in regulating numerous ecological and evolutionary processes (Johnson *et al*., 2012; Siefert *et al*., 2015; Des Roches *et al*., 2018).

To better understand how defoliation impacts plant–soil interactions, biotic interactions among plants and soil microorganisms as well as between neighbouring plants need to be considered. For example, root exudates can enhance the decomposition of organic matter through priming effects but also can slow down litter and organic matter decomposition (Kuzyakov *et al*., 2000; Saar *et al*., 2016; Wang *et al*., 2016; Panchal *et al*., 2022; Heredia‐Acuña *et al*., 2023). The underlying causes are still debated but likely depend on the chemical composition and relative quality of root exudates and dead organic matter. Second, plants and soil microorganisms can be both limited by N availability, and hence increased carbon inputs via root exudation could potentially enhance the N demand of soil biota and may not necessarily result in higher N uptake by the plants due to plant–microbial competition (i.e. microbial immobilisation of N; Bardgett *et al*., 2003; Dunn *et al*., 2006). Therefore, the beneficial effect of enhanced root exudation on plant N status depends on the specific microbial groups that the chemical composition of exudates stimulates. For example, if enhanced exudation upon defoliation is indeed an adaptive response to acquire more N, the exudates would preferentially stimulate ammonification but suppress nitrification to minimise energetic costs and reduce competition with nitrifiers (Coskun *et al*., 2017).

In addition to interactions with litter decomposers, defoliation can simultaneously modify interactions with other soil fungi. Defoliation causes a temporary decline in photosynthesis and hence availability of carbon that can be invested belowground. This is clearly seen from reduced allocation to root growth in defoliated plants (Gao *et al*., 2008; Liu *et al*., 2023), although the opposite responses have also been documented (Mcnaughton *et al*., 1998; Ferraro & Oesterheld, 2002). In addition to reduced root growth, plants may also reduce carbon allocation to arbuscular mycorrhizal (AM) fungi, although the effects of defoliation due to grazing on AM fungi are also highly variable (van der Heyde *et al*., 2017; Faghihinia *et al*., 2020; Yang *et al*., 2020; Xu *et al*., 2025). On the contrary, the impact of defoliation on soil pathogens is mostly unknown. Grazing is generally considered to reduce pathogen pressure aboveground as leaves are exposed to pathogens for a shorter time and grazing makes abiotic conditions less favourable for pathogens (Hatcher & Paul, 2000; Liu *et al*., 2021). Leaf damage can potentially also induce systemic changes in plant defences and hence result in either suppressed or enhanced belowground pathogen resistance depending on complex synergies or trade‐offs between defences against above‐ and belowground herbivores and microbial pathogens (Bezemer & Van Dam, 2005; De Vos *et al*., 2006). Lastly, plant–herbivore interactions are known to vary between plant species and to be affected by species richness, with cascading effects on vegetation dynamics and ecosystem functions (Liu *et al*., 2015; Sheng *et al*., 2023; Abrigo *et al*., 2024). Plant species are known to have complementary effects on plant productivity and soil microbial processes, and these effects can be either enhanced or suppressed in the presence of grazing (Kotowska *et al*., 2010; Barthelemy *et al*., 2017; Eskelinen *et al*., 2022). However, little is known about the importance of intraspecific plant diversity in modulating plant and soil responses to defoliation. Higher genotypic diversity has been shown to enhance temporal stability in plant productivity and result in higher inputs of carbon into soil via root exudation due to complementary in plant functional traits (Prieto *et al*., 2015; Semchenko *et al*., 2021). As plant genotypes also differ in their effects on soil processes and responses to herbivore damage (Bowers & Stamp, 1993; Noy‐Meir & Briske, 2002; Schweitzer *et al*., 2008), functional complementarity among genotypes may modulate plant population responses to defoliation and the distribution of resources between plants and soil communities. Therefore, to fully understand the impact of defoliation on the carbon and N dynamics, biotic interactions between plant genotypes and different microbial guilds, including decomposers, mutualists and pathogens, must be evaluated.

In this study, we exposed genotypic monocultures and mixtures of a common temperate grass *Anthoxanthum odoratum* L. to a defoliation treatment to test how plant intraspecific diversity interacts with defoliation to shape (1) carbon capture and aboveground carbon fluxes, (2) belowground allocation of carbon via root exudation, and cascading effects on (3) litter decomposition, soil enzymatic activity, and partitioning of mineralised N between plants and microbial biomass, and (d) soil fungal community diversity and composition.

We hypothesised that defoliation will first have a negative impact on photosynthesis but enhance root exudation, litter decomposition, N mineralisation and N uptake in the process of recovery from defoliation, which should compensate for the removed leaf biomass (Hamilton *et al*., 2008; Sun *et al*., 2017). We also expected that defoliation will reduce the relative abundance of soil pathogens due to systemic responses to aboveground damage and suppress relative abundance and diversity of mycorrhizal fungi due to the enhanced root exudation and leaf regrowth coming at the expense of carbon investment into mycorrhizal symbiosis. We hypothesised that higher genotypic diversity would enhance productivity, root exudation and microbial activity and would modulate the effects of simulated grazing on carbon and N dynamics. In particular, we predicted that the negative effects of defoliation on photosynthesis will be dampened and the ability of the plant population to recover from defoliation via enhanced N uptake will be enhanced in genotypic mixtures due to functional complementarity among plant genotypes (Prieto *et al*., 2015; Schöb *et al*., 2015; Semchenko *et al*., 2021). Finally, we also expected that genotypic monocultures would be more susceptible to soil pathogen accumulation compared with mixtures due to more pathogen‐resistant genotypes supressing pathogen spread in the mixtures, as has been shown for plant species richness, which reduced pathogen load (Mitchell *et al*., 2002; Liu *et al*., 2016; van Ruijven *et al*., 2020).

## Materials and Methods

### Experimental design

Twenty *Anthoxanthum odoratum* L. genotypes were collected from the Park Grass Experiment, Rothamsted Research Station, UK. Park Grass is the longest‐running fertilisation experiment in the world, and genetic and phenotypic differentiation between *A. odoratum* individuals across different fertilisation treatments has been previously investigated at the site (Silvertown *et al*., 2006). Plants were collected from two unfertilised and two fertilised plots to capture genetic diversity present at the site and at least 1 m apart each other within each plot to ensure that each individual represented a distinct genotype (*A. odoratum* is self‐incompatible and does not spread clonally at long distances). Ten genotypes from fertilised and 10 from unfertilised plots were used in this study to create diverse genotype mixtures, but fertilisation history was not directly considered in this study. In unperturbed (unclipped) conditions, genotypes showed 1.7–3 times variation in key traits (Table S1; SLA: 0.18–0.30 cm^2^ mg^−1^; SRL 26–52 cm mg^−1^), productivity (shoot mass 2.6–5.2 g) and soil microbial biomass (0.527–1.620 mg C g dry soil^−1^), which is comparable to variation observed among temperate grassland species with contrasting growth strategies (Liu *et al*., 2023; 1.4–4.5 times variation: SLA 0.13–0.37 cm^2^ mg^−1^; SRL 5.15–19.3 cm mg^−1^; shoot mass 0.44–1.96 g, microbial biomass 0.176–0.253 mg C g dry soil^−1^), but also comparable to intraspecific variation found in other grass species (Buchmann *et al*., 2018; Roybal & Butterfield, 2019).

**Fig. 1:**
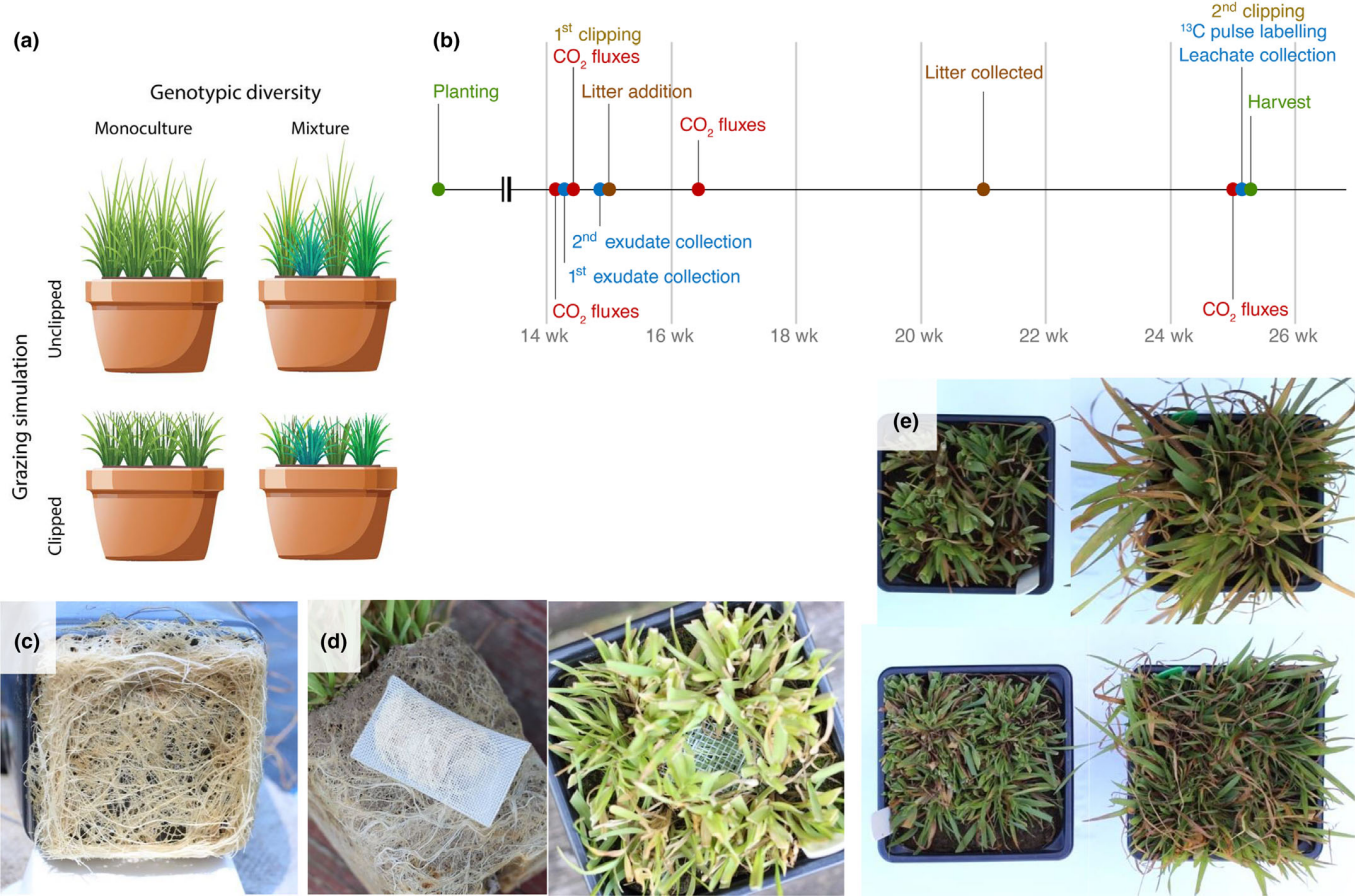
Experimental design. Twenty genotypes of *Anthoxanthum odoratum* were grown as either monocultures or random mixtures of four genotypes and were either subjected to simulated grazing (defoliation after 14 and 25 wk of growth) or left unclipped, resulting in 80 microcosms in total (a, b). Root exudates were collected a day before and 3 d after the first defoliation event from a chamber underneath the pots where roots were accumulated ((c) shows roots when chamber is uncovered). After 15 wk of growth, ^15^N‐labelled leaf and root litter was inserted into the pots and was incubated for 6 wk before removing (d). Carbon fluxes were measured 2 d before, immediately after, 2 wk and 10 wk after the first clipping event. The day before the final harvest, the plants were labelled with ^13^CO_2_ for 2 h, and root exudation was assessed as ^13^C detected in soil leachates at 4 h after the start of pulse labelling. Plants were harvested 24 h after pulse labelling. (e) Depicts two examples of genotypic monocultures (clipped and unclipped) to demonstrate variation in growth form. N, nitrogen.

The genotypes were propagated vegetatively in a glasshouse for the first year and in an outdoors common garden for the second year before the start of the experiment. During the first year, plants were replanted into fresh compost (No. 1; William Sinclair Horticulture Ltd, Lincoln, UK) after 3 months and were clipped and fertilised monthly; no further fertilisation and clipping were applied during the second growing season outdoors. In March 2018, individual cuttings of each genotype were planted into pots (8 × 8 × 9.4 cm) filled with 530 g of homogenised soil from an extensively managed grassland immediately adjacent to the Park Grass Experiment (Paleudalf soil, pH 5.6–5.8, well drained, unfertilised, and silty clay loam). Plants were distributed between two genotypic diversity treatments: 20 unique monocultures, each containing four individuals of the same genotype, and 20 unique mixtures, each containing four different genotypes randomly selected from the pool of 20 genotypes and restricted such that each genotype was used in four mixtures. Each unique monoculture and mixture were replicated twice resulting in 80 pots in total. An additional set of four pots were planted with a random selection of genotypes to be used as unlabelled controls to estimate the natural abundances of ^13^C and ^15^N in collected samples; five additional pots were left unplanted as controls for soil fungal community analysis. After growing for 6 wk in a glasshouse, the pots were moved into a common garden outside for the remainder of the experiment. One replicate of each of 20 monocultures and 20 genotypic mixtures were subjected to a clipping treatment in mid‐June (after 14 wk of growth) and immediately before the final harvest in September (25 wk of growth), simulating the traditional mowing regime used in the Park Grass Experiment (Silvertown *et al*., 2006; Fig. 1). Vegetation was clipped to the height of 2 cm, clippings were collected, dried at 40°C and weighed. Pot positions were randomised every 2 wk to avoid location effects. Clipping height was chosen based on preliminary tests that showed that this level of clipping resulted in successful plant regrowth to original size within *c*. 2 months.

### Root exudate measurements

To allow *in situ* collection of root exudates in the absence of soil solution, a plastic container was fitted tightly to the bottom of each pot creating a 40‐ml chamber where roots could grow into through the eight openings at the bottom of the pot. As such, roots in the chamber were physiologically connected to intact shoots and root system experiencing natural soil environment but were kept clean of soil particles, and root exudates could be collected in a controlled manner (Fig. 1c). Root exudates were collected the day before and 3 d after the first clipping event. The chambers at the bottom of the pots were removed, roots rinsed with CaCl_2_ solution (0.66 mg CaCl_2_ l^−1^ MilliQ water, simulating rainwater; Noble *et al*., 2017) and placed into a new, sterile chamber filled with 20 ml CaCl_2_ solution. Roots were left in contact with the solution for 90 min; the solution was collected and another 20 ml of CaCl_2_ solution was added and collected after another 90 min. Root exudates were collected in two steps to reduce the exposure of exudates to microbial decomposition during collection. All collected exudates were immediately filter‐sterilised using 0.22‐μm syringe filters (Millex; Merck Millipore Ltd, Cork, Ireland). Total organic carbon (TOC) in the exudate samples was measured using a total carbon analyser (TOC‐L; Shimadzu, Kyoto, Japan). The plants were not watered immediately before or during root exudate collection to avoid contamination with soil leachates. After root exudate collection, the roots in the chamber were clipped, dried at 40°C, and weighed.

### Litter decomposition

In order to create ^15^N‐labelled material, an additional 20 pots were planted with monocultures of five genotypes of *A. odoratum* (not used in the main experiment). The pots were filled with 1 : 1 sand and soil mixture (same as used in the main experiment) and were fertilised at the rate of 20 kg N ha^−1^ twice (22 May and 1 June) with ^15^NH_4_^15^NO_3_, which was regarded sufficient to effective plant labelling based on previous trials. These plants were harvested after 14 wk of growth (18 June). The soil was gently washed of the roots, and leaf and root material were air‐dried at room temperature for 4 d, cut into 1 cm segments, and homogenised. Each pot of the main experiment then received 0.3 g of ^15^N labelled dried leaf and root material (17.5 and 7.9 atom %, respectively) in two separate positions. Leaf material was placed on the soil surface in the middle of each pot to ensure maximum contact surface between soil and leaf material and covered by a 2.5 × 2.5‐cm mesh square (2‐mm mesh size) held in place with staples to prevent movement or export. Root litter was placed into sealed 3 × 5 cm bags with a 0.5‐mm mesh size. A vertical cut was made into the soil from the bottom of the pot, and the litter bag was inserted into the gap. The root and leaf material was aimed to simulate litter inputs with a caveat that we could not use senescent material to ensure sufficient isotope tracer concentrations in the simulated litter. The litter was inserted into the pots after 15 wk of growth and 4 d after the first clipping event (26 June). After 6 wk of decomposition, the litter was removed, cleared of soil particles, oven‐dried at 40°C until constant weight, and weighed to calculate the proportion of litter remaining.

### Carbon flux measurements

Carbon fluxes were measured four times during the experiment to assess net ecosystem exchange (NEE), respiration and photosynthetic carbon fixation: 2 d before, immediately after, 2 and 10 wk after the first clipping event. To measure the NEE (the sum of photosynthesis and respiration with more negative values indicating net carbon storage and positive values indicating carbon loss), plants were placed inside a sealed, transparent plastic 2.22‐l chamber (90% transparency) and fluxes were measured for 2 min using an infrared gas analyser (EGM4; PP Systems, Amesbury, MA, USA). The plants were then placed in the same chamber covered with foil to exclude light, and total (plant and soil) respiration was measured. Photosynthetic rate was calculated by subtracting respiration from NEE; values were then multiplied by minus one, so that more positive values indicate greater photosynthetic rates. Photosynthetically active radiation (PAR) levels were recorded at three time points during each measurement with a PAR meter (Skye Instruments, Llandridod Wells, UK) and mean PAR levels were calculated and used as a covariate in subsequent analyses. The mean PAR levels for the 4 d of flux measurements were: 1660, 1810, 1660, and 410 μmol m^−2^ s^−1^. Therefore, photosynthesis was probably limited by light availability on the last day of measurements but not on the other 3 d.

### ^13^C labelling

The day before the final harvest, the plants were labelled with ^13^CO_2_: all pots (except five additional pots that were not used in the main experiment and were used as unlabelled controls) were placed simultaneously into a transparent plastic chamber (100 × 100 × 40 cm) and ^13^C enriched air (350 ppm CO_2_, 99 Atom%) was circulated in the chamber for 2 h (mean PAR 1400 μmol m^−2^ s^−1^ outside, 90% transmission through the chamber). The plants from the clipping treatment were clipped to 2 cm immediately after the end of labelling, and the clippings were collected, dried and weighed as described above. Two hours after the end of labelling, the pots were watered to 660 g to bring all pots to nearly 100% water holding capacity, an additional 50 ml of tap water was added, and leachates were collected from the bottom of the pots. TOC and concentrations of ^12^C and ^13^C in the leachates were measured by cavity ring‐down spectrometry using a Picarro G2201‐i δ^13^C‐CO_2_ analyser (Picarro, Santa Clara, USA) coupled with an Aurora 1030 W TOC analyser (OI Analytical, College Station, USA). In the following text, we will refer to root exudates when presenting data on solutions collected from a root chamber underneath the pots, while we refer to soil leachates when presenting data on soil solutions collected after pulse labelling. Soil leachates collected from labelled pots were enriched in ^13^C (δ^13^C ranged from −27.9 to 9.7) compared with an unlabelled control pot (δ^13^C = −30.8). The concentration of ^13^C in the leachate solution was calculated by multiplying the total carbon concentration measured with the TOC analyser by the ^13^C atom proportion of the sample. The total amount of ^13^C in the leachates (in μg) was calculated by multiplying the concentration of ^13^C in the leachate solution by the total volume of leachate collected.

### Final harvest

Twenty‐four hours after the labelling, shoots were removed from each pot. A subsample of soil was collected from each pot for soil chemical and microbial analysis *c*. 48 h after the labelling, and roots were carefully removed from soil by washing. A subsample of leaves was stored upright in sealed plastic bags with leaf bases submerged in water at 4°C overnight, and rehydrated leaves were weighed. The leaf samples and a sample of roots were scanned with an Epson Expression 11000XL scanner (Epson, Tokyo, Japan). Leaf area and root length, diameter and volume were determined using the WinRHIZO software (Régent Instruments Inc., Québec, QC, Canada). All plant material was dried at 40°C to constant weight and weighed. Leaf dry matter content (LDMC) was calculated as the ratio of leaf dry mass divided by leaf fresh mass; specific leaf area (SLA) was calculated as the leaf area divided by dry weight; specific root length (SRL) was calculated as root length divided by root dry mass; root tissue density was calculated as root dry mass divided by root volume.

To measure plant available N, 5 g fresh weight of soil was weighed into a container and 25 ml 1 M KCl was added to each bottle. Containers were shaken on an orbital shaker for 1 h at 200 rpm. Samples were filtered through 11 μm medium flow filter paper (Whatman 42) and analysed for NO_3_^−^ and NH_4_^+^ using a SEAL AA3 autoanalyser (Seal Analytical Ltd, Fareham, Hampshire, UK). To measure microbial biomass carbon and N, 5 g fresh weight soil was extracted with 25 ml 0.5 M K_2_SO_4_ by shaking on an orbital shaker for 30 min at 200 rpm, and another 5 g was fumigated with chloroform for 24 h and extracted with the same method. The samples were filtered through 11 *μ*m medium flow filter paper, frozen at −80°C and freeze‐dried. Milled leaf and root samples and freeze‐dried K_2_SO_4_ extracts were analysed for total C and N, and ^13^C and ^15^N isotopes, using an Elementar Vario EL Cube or Micro Cube elemental analyser (Elementar Analysensysteme GmbH, Hanau, Germany) interfaced to a PDZ Europa 20‐20 isotope ratio mass spectrometer (Sercon Ltd, Cheshire, UK; performed by UC Davis Stable Isotope Facility, USA). For carbon and N, respectively, the final delta values are expressed relative to international standards VPDB (Vienna PeeDee Belemnite) and air. Soil microbial biomass was calculated as the difference in N and carbon content between nonfumigated and fumigated samples per unit of dry mass using extraction efficiency values of K_EC_ 0.35 and K_EN_ 0.54 for microbial C and N, respectively (Brookes *et al*., 1985; Sparling *et al*., 1990). To measure residual ^13^C in soil solution 48 h after pulse labelling, an additional 5 g of soil was extracted with MilliQ water and ^13^C and TOC were measured as described for leachates in the ^13^C labelling section above. Residual ^13^C per unit of dry soil was calculated by multiplying the concentration of ^13^C in the soil extract by the extraction volume (25 ml) and dividing by dry mass of extracted soil. The comparison between natural abundances of ^13^C and ^15^N in unlabelled pots and enrichment in labelled pots is presented in Table S2. To determine soil moisture content, an additional soil sample was weighed fresh and after drying at 105°C to constant weight.

### Soil enzymatic activity

We measured the potential activity of eight soil enzymes that catalyse key C‐, N‐, and P‐cycle processes. β‐glucosidase (GLC), cellobiohydrolase (CBH), xylosidase (XYL), N‐acetylglucosaminidase (NAG) and acid phosphatase (AP) were measured photometrically according to Jackson *et al*. (2013), with the modifications detailed in Cordero *et al*. (2023). Urease (URE) was measured as described in Cordero *et al*. (2019). Phenoloxidase (POX) and peroxidase (PER) activities were measured following Sinsabaugh & Linkins (1988) with the modifications described in Cordero *et al*. (2023). In all cases, the activities were measured at room temperature (18°C), pH equivalent to soil pH (pH = 5.0) and under saturating substrate concentration. The concentration of end products of the reactions was measured by its absorbance using a plate reader (CLARIOstar; BMG Labtech, Ortenberg, Germany). See Table S3 for further details.

### ITS sequencing of fungal communities and assessment of root colonisation

DNA was extracted from 250 mg of freeze‐dried soil samples, with the PowerSoil DNA isolation Kit (Qiagen). Amplification of ITS2 was done with the primer pair gITS7ngs and ITS4ngsUni (Tedersoo & Lindahl, 2016; Nilsson *et al*., 2019). PCR was carried out in a final 25 μl volume with reaction buffer 1× (KAPA HiFi buffer), 0.3 mM of KAPA dNTP mix, 0.6 μM of gITS7ngs primer, 0.3 μM of ITS4ngsUni primer, and 0.5 U of KAPA HiFi Hotstart DNA polymerase (Roche). Negative controls with ultra‐pure water were included in each PCR run to detect possible contaminations. The PCR temperature profile was: initial denaturation at 95°C for 3 min, followed by 30 cycles of denaturation at 98°C for 20 s, annealing at 53°C for 30 s, and elongation at 72°C for 30 s, and a final elongation step at 72°C for 1 min. Amplifications were performed in a thermal cycler (SimpliAmp; Applied Biosystems, Waltham, MA, USA) and PCR products analysed by electrophoresis on 1.5% agarose TBE gels dyed with GelGreen (Biotium Inc., Fremont, CA, USA). Amplicons were then purified with AMPure XP magnetic beads (Beckman Coulter, Inc., Brea, CA, USA). Library preparation and sequencing was carried out as external service (Earlham Institute, UK), on a MiSeq v2 with 250 bp pair‐end reads (Illumina, Inc., San Diego, CA, USA). Alongside the samples, a mock community sample of 19 strains' genomic DNA even mix was included (Bakker, 2018).

Sequences were analysed using the DADA2 pipeline (Callahan *et al*., 2016) with default parameters, after removing primers using cutadapt (Martin, 2011). After filtering and de‐noising steps 61% of the reads were retained, and 12 912 amplicon sequence variants (ASV) were identified. Taxonomic identification was performed with the dada2::assignTaxonomy function, using the UNITE reference database (sh_general_release_dynamic_all_04.04.2024.fasta). The data were refined by three consecutive steps: (1) cleaned to counteract the problem of cross‐talk with the UNCROSS2 function (Edgar, 2018), which estimated a 0.0098 cross‐talk rate (estimated *de novo*) and removed 1037 reads (0.018% of reads) from 18 ASVs; (2) Lulu algorithm to reduce the number of erroneous ASVs and achieve more realistic biodiversity metrics (Frøslev *et al*., 2017), setting the minimum match at 99.5%, and the rest parameters set as default, which merged 108 ASVs. The election of these parameters was based on the results of the mock community sample; (3) minimal abundance filtering, by only keeping ASVs that had a relative abundance > 0.005% in at least one sample. This step removed 2392 ASV which accounted for 6932 reads (0.12% of reads); (4) finally, only fungal sequences were retained. The final database contained 4353 ASVs and 2866 876 reads. Sampling depth varied from 21 385–57 438 (mean 36 207) reads, except for one sample with sequencing depth of 6541, which was removed from the analysis. Sequencing depth did not differ significantly between treatments. Rarefaction curves were plotted using the *rarecurve* function in vegan (Oksanen *et al*., 2024), and these indicated that sampling depth was sufficient for all samples. ASVs were assigned to two functional guilds – plant pathogens and saprotrophs – based on their primary lifestyle in the FungalTraits database v.1.2 (Põlme *et al*., 2020). AM fungi were identified based on taxonomy (belonging to phylum *Glomeromycota*). To confirm that ASVs identified as pathogens and AM fungi were responsive to the studied plant host (*A. odoratum*), ASV relative abundance in experimental microcosms was compared with the presence and abundance in control, unplanted microcosms.

The interpretation of molecular analysis of AM fungal communities in soil was supported by visually assessing colonisation of roots by AM fungi in five replicates from four different treatments, namely unclipped monoculture, unclipped mixture, clipped monoculture, and clipped mixture. Dried roots were cleared and stained with ink, and colonisation quantified using the gridline intersect method (McGonigle *et al*., 1990).

### Statistical analysis

All statistical analyses were done in R v.4.5.2 (R Core Team, 2025). Linear mixed models implemented in the R package lme4 (Bates *et al*., 2015) were used to evaluate the effects of clipping and genotypic diversity (both fixed‐effect factors with two levels each) and their interaction on plant total biomass, root to shoot biomass ratio, carbon fluxes, root exudation, ^13^C quantity in soil leachates, soil ammonium and nitrate availability, litter decomposition rate, soil microbial biomass C and N, and soil enzymatic activity. Genotypic composition of the pot was included in the models as an intercept‐level random term as two pots with identical genotypic composition were included in the design (one pot clipped and one pot left unclipped). The response variables were log‐transformed (natural logarithms) whenever necessary to satisfy the assumptions of normality and homogeneity of variance in model residuals, which were tested using the DHARMa R package (Hartig, 2024). ANOVA tests were subsequently performed on each linear mixed model using the car R package (Fox & Weisberg, 2019). Mixed models for carbon fluxes included PAR availability as a covariate to account for potential variation due to differences in light levels.

To examine isotope partitioning between plants, exudates, and soil microbial biomass, we calculated a ratio between ^13^C in microbial biomass per gram of dry soil to the amount of ^13^C in soil leachates (i.e. proportion of ^13^C in leachates that is retained in microbial biomass), as well as a ratio of ^15^N in microbial biomass per gram of dry soil to ^15^N atom% in leaves (i.e. relative partitioning of ^15^N between microbial biomass and plants). In the latter, we decided to use ^15^N enrichment in sampled leaves in the calculation rather than extrapolate to total shoot mass, as shoot stems and senescent leaves likely had different ^15^N enrichment. We also calculated a ratio of ^13^C in soil leachates to ^13^C in leaves clipped at the end of labelling to assess the proportion of recently fixed C allocated to exudation.

To examine the relationships between root exudation and decomposer functions, linear mixed models were fitted with soil enzymatic activity or ^15^N concentration in leaves as the response variables and ^13^C quantity in soil leachates as a predictor; clipping treatment was added as a fixed factor in the models, while the diversity treatment was not included as it was collinear with ^13^C quantity.

Linear mixed models were also applied to examine the effects of clipping and genetic diversity on the relative abundances, richness, and diversity of fungal communities. Species diversity was quantified using the Shannon index, calculated from abundances of observed ASVs in each sample with the R package vegan (Oksanen *et al*., 2024). Each model included an interaction between clipping and genetic diversity as fixed factors, sequencing depth as a covariate to control for variation in sequencing effort, and the genotypic composition of the pot as an intercept‐level random term. To provide a more comprehensive functional perspective, this modelling approach was applied to key fungal groups: all fungi, pathogens, AM fungi, and saprotrophs. Additional models were fitted for pathogens after excluding the numerically dominant ASV11 (ASV11 was highly responsive to clipping and was not contamination as it was absent from unplanted control pots), as well as for substrate‐specific subgroups of saprotrophs, namely wood saprotrophs, litter saprotrophs, and soil saprotrophs. As above, ANOVA tests were conducted for each mixed model, and the assumptions regarding model residuals were verified using the DHARMa package (Hartig, 2024).

The effects of clipping and genetic diversity on fungal community composition were first evaluated using permutational multivariate analysis of variance (PERMANOVA; Anderson, 2001; McArdle & Anderson, 2001) in the vegan R package. Raw abundances for each fungal group (i.e. all fungi, pathogens, AM fungi, all saprotrophs, wood saprotrophs, litter saprotrophs, and soil saprotrophs) were standardised to relative abundances using the function decostand in vegan. Bray–Curtis dissimilarity matrices were then computed and used as input for PERMANOVA models. Before model fitting, homogeneity of group dispersion was assessed using the PERMADISP2 method (Anderson, 2006; Anderson *et al*., 2006) in vegan. Each PERMANOVA model included an interaction between clipping and genetic diversity as independent variables, and the ‘lingoes’ correction was applied to prevent negative eigenvalues during the modelling process (Legendre & Andersson, 1999). Statistical significance was determined via 1000 permutations. To visualise the compositional distribution of fungal communities, principal coordinate analysis (PCoA) with the ‘lingoes’ correction was performed on each Bray–Curtis distance matrix using the ape R package (Paradis & Schliep, 2019). To further examine which fungal saprotroph and mycorrhizal families and pathogen genera showed differential abundance patterns across treatments, we fitted general linear models with a negative binomial distribution and tested the significance of treatment effects with Wald test and applied false discovery rate correction of *P*‐values using DESeq2 R package (Love *et al*., 2014).

## Results

### CO_2_ fluxes and plant biomass

**Fig. 2:**
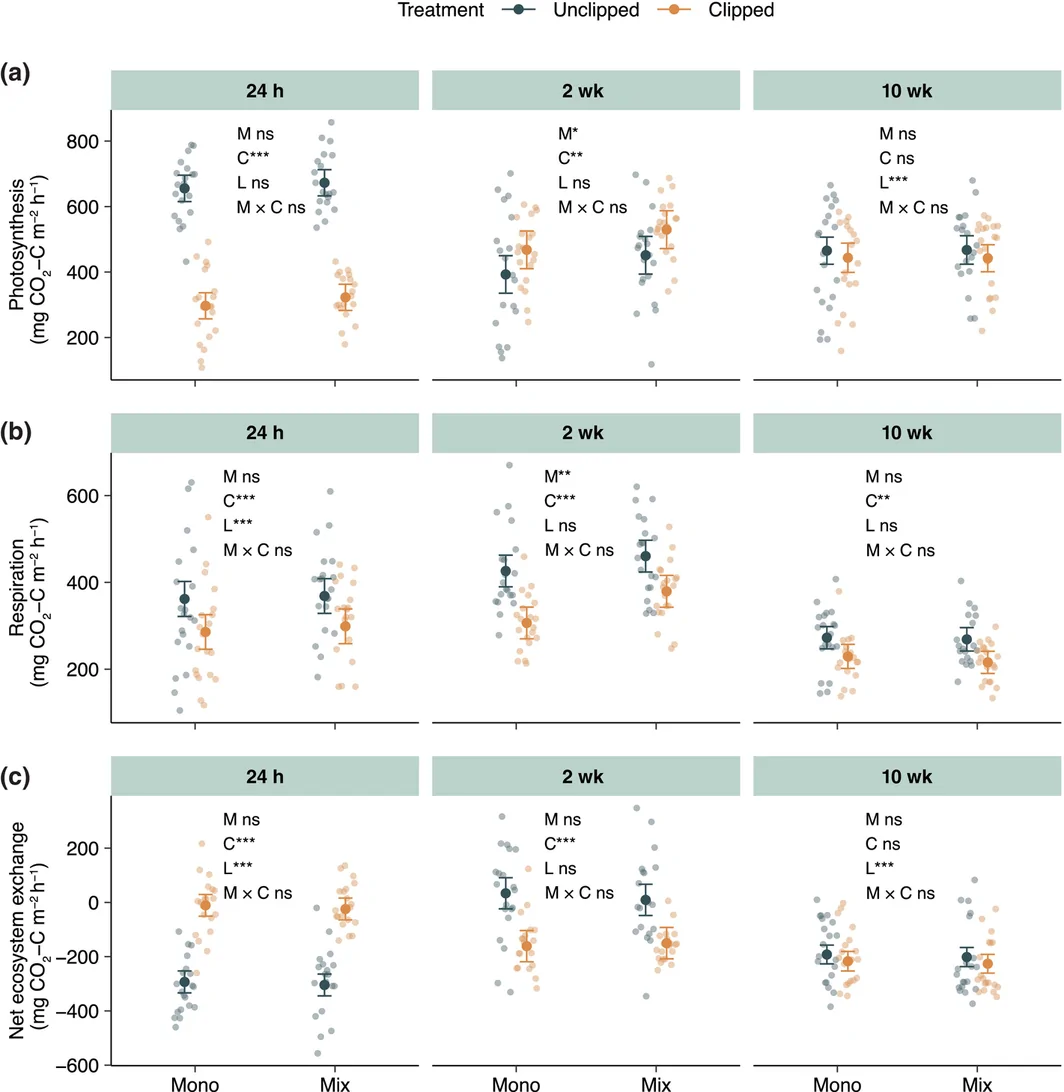
Effect of plant genotypic diversity (*Anthoxanthum odoratum*) and defoliation treatment on microcosm carbon fluxes 24 h, 2 wk, and 10 wk after a leaf clipping event. (a) Photosynthesis, (b) respiration, (c) net ecosystem exchange. Control plants that were left unclipped are shown in grey, and plants that were clipped to 2 cm height are shown in orange. Negative values of net ecosystem exchange indicate net carbon storage and positive values net carbon loss. Predicted means and 95% confidence intervals are shown. The significance of model terms is shown as follows: C, effect of clipping; L, effect of light availability; M × C, interactive effect of genotypic diversity and clipping; M, effect of genotypic diversity (monoculture or a mixture of four genotypes); ns, *P* > 0.05; *, *P* < 0.05; **, *P* < 0.01; ***, *P* < 0.001.

Twenty‐four hours after a clipping event, we detected a strong negative effect of clipping on photosynthesis (*F*_1,38_ = 303.4, *P* < 0.001), total respiration (*F*_1,38_ = 17.2, *P* < 0.001), and net carbon storage (i.e. NEE shifted from negative to positive, *F*_1,38_ = 220.5, *P* < 0.001), but no significant effect of genotypic diversity on those carbon fluxes (Fig. 2; Table S4). Two weeks following the clipping event, microcosms that experienced clipping showed enhanced photosynthesis but reduced respiration, which translated into higher net C storage in the clipped than unclipped treatment (Fig. 2; Table S4). Genotypic mixtures were characterised by significantly higher metabolic activity at this time point (both in terms of photosynthesis, *F*_1,37_ = 4.28, *P* = 0.046, and respiration, *F*_1,37_ = 8.37, *P* = 0.006; Fig. 2; Table S4). By 10 wk after clipping, clipped microcosms were showing significantly lower respiration but similar photosynthesis as unclipped microcosms (Fig. 2; Table S4).

**Fig. 3:**
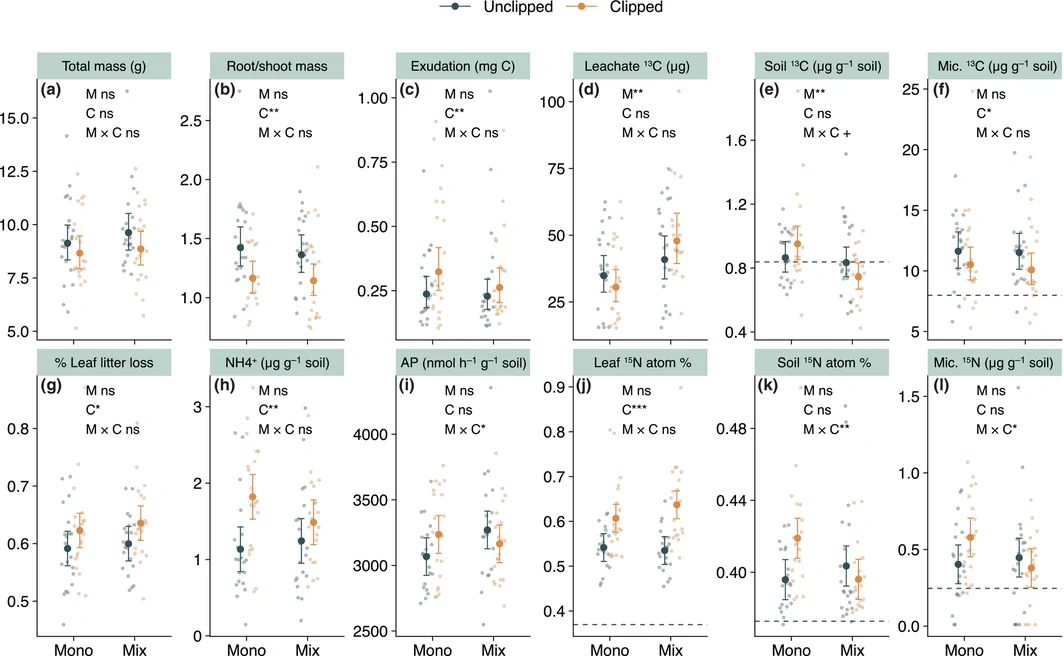
Effect of plant genotypic diversity (*Anthoxanthum odoratum*) and defoliation treatment on plant and soil characteristics. (a) Total plant dry biomass, (b) root to shoot biomass ratio, (c) dissolved organic carbon detected in root exudates 3 d after clipping, (d) soil leachate ^13^C, 2h after labelling, (e) dissolved soil organic ^13^C, 48 h after labelling, (f) soil microbial biomass ^13^C, (g) proportion of leaf litter decomposed, (h) ammonium availability in soil, (i) activity of acid phosphatase, (j) leaf ^15^N, percentage of ^15^N atoms, (k) ^15^N in soil solution, percentage of ^15^N atoms, (l) soil microbial biomass ^15^N. Unclipped plants are shown in grey, and clipped plants in orange. Predicted means and 95% confidence intervals are shown. The significance of model terms is shown as follows: C, effect of clipping, M, effect of genotypic diversity (monoculture or a mixture of four genotypes); M × C, interactive effect of genotypic diversity and clipping; ns, *P* > 0.1; +, *P* < 0.1; *, *P* < 0.05; **, *P* < 0.01; ***, *P* < 0.001. Dashed horizontal lines indicate natural abundance of isotopes in unlabelled samples. N, nitrogen.

By the end of the experiment, clipped and unclipped microcosms accumulated similar total biomass (this includes removed biomass for the clipped treatment), but clipped microcosms significantly reduced relative allocation to root biomass (Fig. 3a,b; Table S5). Genotypic diversity did not significantly affect either total biomass production (*F*_1,38_ = 0.53, *P* = 0.472) or the amount of biomass removed during clipping (*F*_1,38_ < 0.1, *P* = 0.959). Most of the root and leaf traits were not significantly affected by treatments except for leaf N content, which was higher in clipped plants (Table S5). Genotypic mixtures showed significantly higher variation in plant height among individuals within a microcosm compared with genotypic monocultures (Table S5; mean coefficient of variation for plant height in monocultures and mixtures was 14 and 22% before clipping and 9 and 18% at the end of the experiment).

### Belowground carbon distribution

Three days after the first clipping event, a significant increase in root exudation was detected in clipped microcosms (*F*_1,38_ = 9.55, *P* = 0.004; Fig. 3c; Table S6). At the end of the experiment and 2 h after the second clipping event, no significant increase in root exudation was detected in response to clipping (Table S6). Instead, we detected significantly higher root exudation in genotypic mixtures than monocultures, detected as ^13^C in soil leachates (*F*_1,38_ = 7.84, *P* = 0.008; Fig. 3d; Table S6). The amount of ^13^C recovered in soil leachates of genotypic mixtures was on average 36% higher than that in genotypic monocultures. Monocultures and mixtures did not significantly differ in biomass productivity (Fig. 3a) nor ^13^C enrichment in leaves at the end of labelling (*F*_1,38_ = 1.04, *P* = 0.314). Hence, mixtures were characterised by 1.5 times higher proportion of photosynthetically fixed ^13^C being recovered in soil leachates (1.0 vs 1.5% of fixed ^13^C detected in leachates within 2 h, *F*_1,38_ = 9.85, *P* = 0.003). Two days after ^13^C labelling, ^13^C in soil solution was on average 15% higher in the genotypic monocultures than mixtures (*F*_1,38_ = 6.21, *P* = 0.017, Fig. 3e; Table S6). Clipping treatment resulted in significantly lower incorporation of exuded ^13^C into soil microbial biomass (*F*_1,38_ = 4.23, *P* = 0.047; Fig. 3f; Table S6). In addition, a significantly lower fraction of ^13^C per unit of exuded ^13^C was incorporated into microbial biomass in genotypic mixtures than monocultures (*F*_1,38_ = 6.44, *P* = 0.015; Fig. S1a).

### Decomposer activity and nitrogen cycling

The decomposition of both leaf and root litter, and ammonium availability in soil, was enhanced by clipping (Fig. 3g,h; Table S7). Clipped plants also had a significantly higher concentration of litter‐derived ^15^N in their leaves compared with unclipped plants (*F*_1,38_ = 33.5, *P* = 0.001; Fig. 3j). Clipping and diversity treatments had interactive effects on the availability of ^15^N in the soil solution and on the microbial biomass ^15^N (Fig. 3k,l; Table S7). Clipping enhanced ^15^N availability in soil and microbial biomass ^15^N in the genotypic monocultures but not in the genotypic mixtures (*F*_1,38_ = 7.87, *P* = 0.008 and *F*_1,38_ = 4.75, *P* = 0.036, respectively). The relative retention of ^15^N in microbial biomass compared with leaf biomass in clipped microcosms was, on average, 11% lower in genotypic mixtures than in monocultures (*F*_1,38_ = 5.02, *P* = 0.031; Fig. S1b).

Soil enzyme activities were affected by clipping in divergent ways in monocultures and mixtures. Phosphatase activity was enhanced by clipping in genotypic monocultures but reduced in mixtures (interactive effect *F*_1,38_ = 4.62, *P* = 0.038; Fig. 3; Table S7). Meanwhile PER and POX enzyme activities tended to be enhanced by clipping only in genotypic mixtures (interactive effect for PER activity *F*_1,38_ = 3.26, *P* = 0.079, and POX activity *F*_1,38_ = 3.43, *P* = 0.072; Fig. S2; Table S7). PER and POX enzyme activities, as well as the concentration of litter‐derived ^15^N in plant leaves, were positively correlated with root exudation as measured by ^13^C in soil leachates, and the relationship tended to be stronger in the clipped treatment (Fig. S3; Table S8).

### Soil fungal community structure

Clipping significantly modified soil fungal community composition, including the composition of pathogen (*R* ^2^ = 0.05, *P* < 0.001), AM fungal (*R* ^2^ = 0.02, *P* < 0.001) and saprotroph communities (*R* ^2^ = 0.03, *P* < 0.001; Fig. S4; Table S9). By contrast, plant genotypic diversity did not significantly affect the community composition of all fungi or any fungal guild (Table S9).

**Fig. 4:**
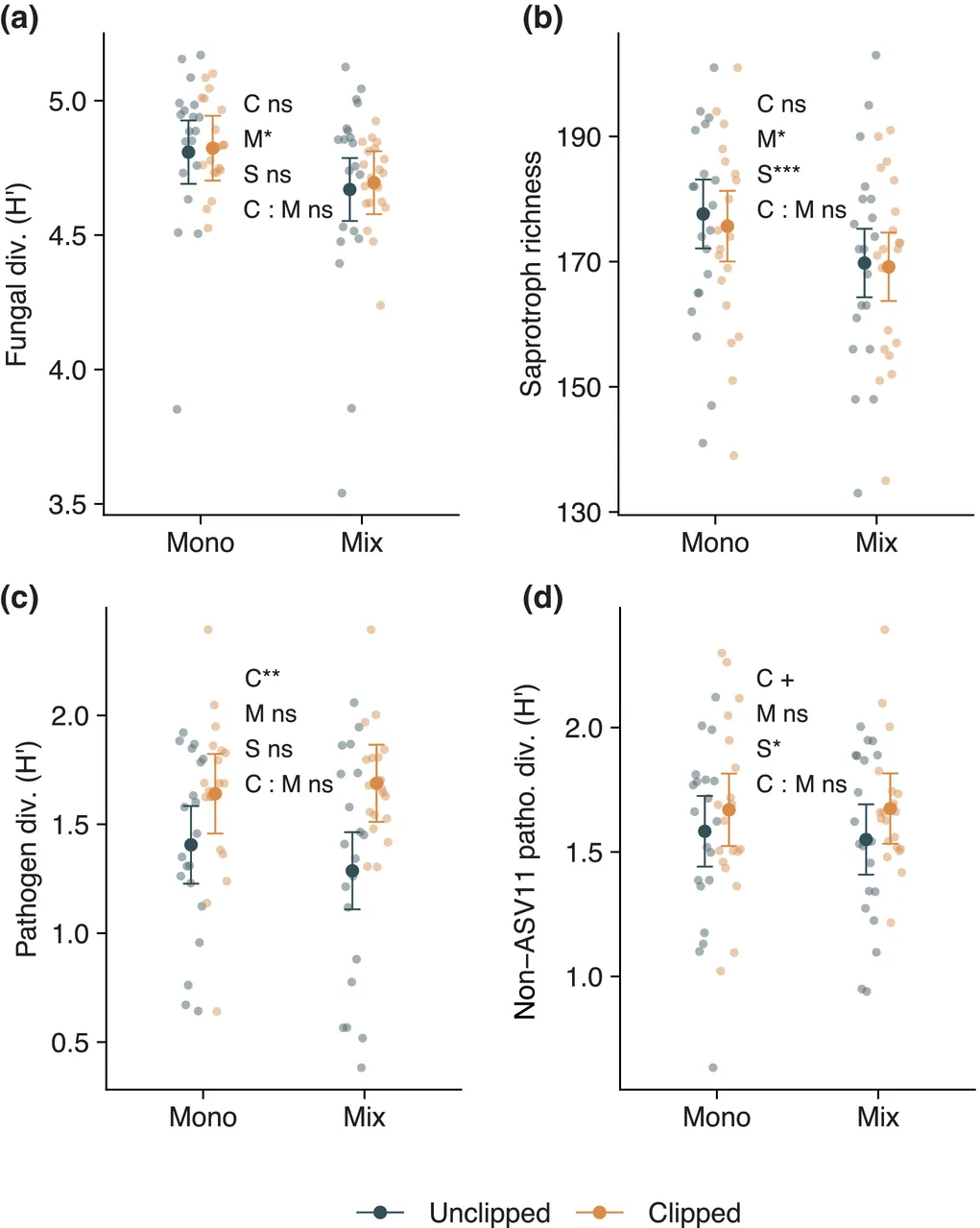
Effect of plant genotypic diversity (*Anthoxanthum odoratum*) and defoliation treatment on Shannon diversity (H') and richness of (a) all soil fungi, (b) saprotrophs, (c) putative pathogens, (d) putative pathogens excluding ASV11 that dominated pathogen communities. Control plants that were left unclipped are shown in grey, and plants that were clipped are shown in orange. Predicted means and 95% confidence intervals are shown. The significance of model terms is shown as follows: C, effect of clipping; M, effect of genotypic diversity (monoculture or a mixture of four genotypes); S, effect of sequencing depth; ns, *P* > 0.1; +, *P* < 0.1; *, *P* < 0.05; **, *P* < 0.01.

The overall richness and diversity of soil fungi was not affected by clipping but was significantly lower in genotypic mixtures than monocultures (Fig. 4a; Table S10). Genotypic mixtures also showed reduced saprotroph richness (Fig. 4b; Table S10). Clipping enhanced the Shannon diversity of putative soil pathogens, but this effect became marginally nonsignificant after excluding the dominant pathogen ASV11, identified as a member of the genus *Eocronartium* (Fig. 4c,d; Table S10). The overall diversity and richness of AM fungi were not affected by the treatments (Table S10).

**Fig. 5:**
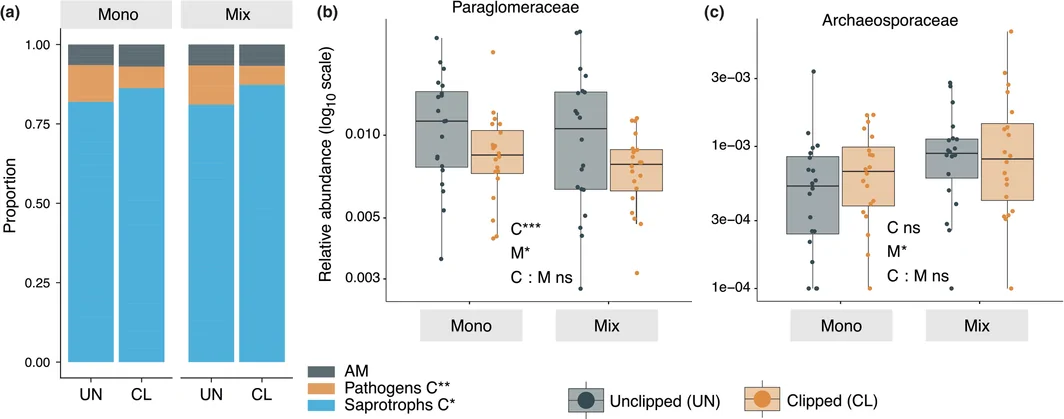
Relative abundance of (a) different fungal guilds among assigned amplicon sequence variants (ASVs) and (b, c) two AM fungal families ((b) – *Paraglomeracea* and (c) – *Archaeosporaceae*) in genotypic monocultures (Mono) and mixtures (Mix) in unclipped (UN) and clipped treatment (CL) of *Anthoxanthum odoratum*. Boxes represent the interquartile range (IQR), the central line indicates the median, and whiskers extend to the largest and smallest values within 1.5 × IQR. Dots represent individual data points. The significance of model terms explaining variation in the relative abundance of each fungal group is shown as follows: C, effect of clipping; M, effect of genotypic diversity; ns, *P* > 0.1; *, *P* < 0.05; **, *P* < 0.01; ***, *P* < 0.001. AM, arbuscular mycorrhizal fungi.

Clipping strongly reduced the relative abundance of putative fungal pathogens: on average, the relative abundance of putative pathogens was 75% higher in unclipped plants compared with clipped plants (*F*_1,37_ = 13.31, *P* = 0.001; Figs 5a, S5a). The ten most common and abundant putative pathogenic ASVs included well‐known generalist pathogens from the genera *Fusarium* and *Alternaria* as well as more grass‐associated genera, such as *Pyrenophora* and *Ophiosphaerella* (Table S11). Out of 135 putative plant pathogen ASVs detected in microcosms planted with *A. odoratum*, 90 ASVs were absent in control unplanted microcosms. However, the effect of clipping on pathogen abundance was mostly driven by the most dominant pathogen ASV11 (*Eocronartium*). When ASV11 was excluded from the analysis, a marginally nonsignificant interaction between clipping and diversity was detected: relative abundance of pathogens was higher in monocultures than in mixtures in the unclipped treatment but was suppressed by clipping in monocultures and enhanced in mixtures (*F*_1,37_ = 3.90, *P* = 0.056; Fig. S5b; Table S12). The analysis of differential abundance of putative pathogen genera only detected significant differences in the genus *Eocronartium*, which was overrepresented in the unclipped compared with the clipped treatment (*P* = 0.026, after false discovery rate (FDR) correction).

Clipping significantly increased the relative abundance of saprotrophs, while no significant effects of plant genotypic diversity were detected (Fig. 5a; Table S12). The analysis of differential abundance of fungal families identified three saprotroph families that responded to treatments: clipping increased the abundance of *Pyronemataceae* both in genotypic mixtures and monocultures, while *Ceratobasidiaceae* and *Hydnodontaceae* increased with clipping in monocultures but not in mixtures (Fig. S6).

The relative abundance of AM fungi was on average 10 times higher in planted than unplanted control microcosms (planted: mean 0.040, range 0.015–0.077; unplanted: mean 0.0038, range 0.0021–0.0046) but was not significantly affected by clipping and diversity treatments (Table S12). Visual assessment confirmed that roots were well colonised and showed that the extent of colonisation did not differ among the four treatments analysed (Fig. S7). However, two families of AM fungi showed differential responses: the relative abundance of *Paraglomeraceae* decreased with clipping and when grown in mixtures compared with monocultures (*P* < 0.001 and *P* = 0.047, respectively, FDR correction), while *Archaeosporaceae* were more abundant in genotypic mixtures than monocultures with no significant response to clipping (*P* = 0.040, FDR correction; Fig. 5b,c).

## Discussion

Simultaneous manipulation of plant genotypic diversity and defoliation showed that defoliation temporarily enhanced photosynthesis and root exudation in both genotypic monocultures and mixtures, which in turn increased litter decomposition, N mineralisation and plant N uptake ensuring compensatory growth. By contrast, genotypic mixtures were characterised by higher metabolic activity (photosynthesis and respiration) and root exudation as well as changes in soil fungal communities, including changes in the relative abundance of some AM fungal families and diversity of saprotrophs. Contrary to the expectation of plant diversity enhancing diversity in other trophic levels, plant genotypic mixtures harboured significantly lower fungal diversity, especially among saprotrophs. Similar findings were obtained from a longer‐term study combining genotypes of several plant species (Johnson *et al*., 2010), suggesting that our results can be generalised to other species and indicating that plant genotypic diversity can serve as a biotic filter on fungal community rather than providing opportunities for niche differentiation. A significant interactive effect of defoliation and plant genotypic diversity emerged when examining the incorporation of mineralised N into microbial biomass and plant tissues, indicating that plant genotypic diversity enhanced the ability of plants to compete with soil microbes for mineralised N.

### Effects on carbon and nitrogen cycling

A defoliation event first suppressed photosynthesis and ecosystem carbon storage, but stimulated root exudation, photosynthesis and carbon storage during a regrowth period, supporting previous studies demonstrating enhanced photosynthetic rates (Iqbal *et al*., 2012) and a short‐term increase in root exudation in response to defoliation (Paterson & Sim, 2000; Hamilton III & Frank, 2001; Hamilton *et al*., 2008). As a result, plants were able to effectively regrow leaf biomass, and no significant difference in total productivity was detected between clipped and unclipped plant groups at the end of the experiment (10 wk after clipping). However, defoliation resulted in reduced allocation to root mass and a moderate reduction in root tissue density, indicating that leaf regrowth came at the expense of investment into root growth and longevity. Reduced root allocation has been observed in previous studies in response to defoliation (Gao *et al*., 2008; Liu *et al*., 2023), although divergent responses have also been reported (Guitian & Bardgett, 2000; Ferraro & Oesterheld, 2002). In addition, defoliation increased the relative abundance of saprotrophs, particularly those from *Pyronemataceae*, and enhanced leaf and root litter decomposition rates, which resulted in higher ammonium but not nitrate availability in the soil. These results indicate enhanced ammonification but not nitrification rates or a fast nitrate uptake by plants or microbes. As a result, higher concentrations of litter‐derived ^15^N were detected in leaves of defoliated plants compared with control plants. Under simulated grazing conditions, positive relationships emerged between recent photosynthates‐derived carbon in root exudates and the activity of oxidative enzymes (degrading lignin among other complex organic compounds), as well as the amount of litter‐derived N in leaves, which provides a mechanistic link for the stimulatory effect of root exudation on the decomposition of soil organic matter (Hamilton *et al*., 2008; Klumpp *et al*., 2009).

Genotypic mixtures showed similar total productivity but increased photosynthesis and whole pot respiration in the middle of the growth season and increased root exudation at the end of the growth season compared with genotypic monocultures. As genotypic mixtures were characterised by higher variation in plant height before clipping and at harvest compared with genotypic monocultures, higher metabolic activity and exudation may be related to complementary resource use in phenotypically diverse plant mixtures, as has been demonstrated in previous studies (Crutsinger *et al*., 2006; Kotowska *et al*., 2010; Semchenko *et al*., 2021). However, higher exudation did not result in higher incorporation of exuded carbon into microbial biomass. Instead, increased plant genotypic diversity was characterised by a lower ratio of ^13^C detected in microbial biomass per unit of ^13^C detected in root exudates, indicating reduced relative carbon incorporation into microbial biomass and greater losses via respiration. Similarly, defoliation resulted in lower microbial carbon despite increased root exudation shortly after clipping. Therefore, higher root exudation was associated with stimulated microbial activity and enhanced N cycling but not increased microbial biomass. This finding contradicts the general trend of root exudate addition resulting in increased microbial biomass (Zhang *et al*., 2025). However, there is accumulating evidence for complex impacts of root exudates on N cycling, microbial biomass turnover and carbon use efficiency, where effects are dependent on the quality of root exudates and background nutrient availability (Spohn *et al*., 2016; Moreau *et al*., 2019; Koyama *et al*., 2025). Our results match observations that root exudation can reduce carbon use efficiency and increase soil enzymatic activities and N mineralisation without changes in microbial biomass (Medina‐Roldán & Bardgett, 2011; Meier *et al*., 2017).

Soil enzymatic activities and N incorporation into microbial biomass were affected by defoliation in different ways depending on the genotypic diversity of plants. Phosphatase activity was increased by clipping only in genotypic monocultures, while oxidative enzymes involved in the degradation of complex organic matter were only enhanced by defoliation in genotypic mixtures and not monocultures. Interestingly, genotypic mixtures also harboured lower richness of saprotrophic fungi compared with monocultures, which may indicate a stronger selective effect of plants on decomposer communities. Plants can alter the chemical composition of their root exudates to recruit specific microorganisms, that allow them to adapt to different nutritional requirements (Zhalnina *et al*., 2018; Zhao *et al*., 2021). Therefore, it may be that altered root exudation in clipped monocultures promoted P‐acquiring organisms reflecting stronger P limitation, while clipped mixtures stimulated fungi capable of oxidative release of tightly bound N, such as in lignin, which may relate to N limitation. The availability of litter‐derived ^15^N in soil solution, and its incorporation into microbial biomass relative to incorporation into plants (i.e. ratio of ^15^N in microbial biomass to that in leaves), was enhanced by defoliation in genotypic monocultures but reduced in genotypic mixtures. This may indicate that plant competitiveness for mineralised N was higher in mixtures than monocultures, which promoted enhanced microbial enzymatic activity but resulted in lower N incorporation into microbial biomass. Competition between plants and soil microbial communities for N is known to be an important regulator of productivity and N retention in grasslands (Bardgett *et al*., 2003).

### Changes in putative fungal pathogen and arbuscular mycorrhizal communities

Defoliation significantly modified the composition of soil fungal communities, including AM fungi, saprotrophs, and putative plant pathogens. Defoliation triggered a drastic decline in plant pathogen relative abundance. Interestingly, the most dominant plant pathogen that experienced the strongest suppression under the defoliation treatment has been described as specific to mosses (genus *Eocronartium*). The high abundance of moss‐specific pathogens is possible as mosses were present in all planted pots. The roots of the studied species also tend to accumulate on the soil surface and intermingle with a moss layer. This pathogen was not detected in any control, unplanted pots. Interactions between bryophytes and vascular plants are a key but often overlooked component of vegetation dynamics (Huber & Kollmann, 2020), and further targeted tests are needed to better understand the observed phenomenon and to ascertain that this pathogen was indeed causing disease in mosses rather than the studied grass species.

When the dominant, possibly moss‐specific, pathogen was excluded from the dataset, only subtle changes in pathogen relative abundance could be detected. The assignment of pathogenic lifestyle to ASVs was done at the genus level, which must be interpreted with caution as pathogenicity can be strain/host‐specific, context‐dependent, and some taxa may have mixed lifestyles. The most dominant and common pathogen ASVs included both well‐known generalists from the *Fusarium* and *Alternaria* genera as well as representatives of grass‐associated *Pyrenophora* and *Ophiosphaerella* (Câmara *et al*., 2000; Marin‐Felix *et al*., 2019). We also found that the majority of pathogenic ASVs were absent in unplanted control microcosms, indicating their accumulation in the plant rhizosphere. However, the analysis of differential abundances did not detect any sensitive taxa besides the strong suppression of *Eocronartium* by clipping. Therefore, based on metabarcoding data, we could not find clear support for the hypotheses for plant genotypic diversity leading to the dilution of host‐specific pathogens and defoliation inducing defences against soil pathogen accumulation.

While the overall relative abundance of AM fungi was not significantly affected by treatments (from both molecular analysis and visual assessment), the analysis of differential abundance of different AM families detected some significant responses. We found that the relative abundance of *Paraglomeraceae*, one of the most abundant AM families in our data, was significantly reduced by clipping and was lower in genotypic mixtures than monocultures. *Paraglomeraceae* is considered a rhizophilic AM guild that invests more of its biomass into occupying roots than exploring soil volume for nutrient foraging (Weber *et al*., 2019). It has also been shown that *Paraglomeraceae* tend to increase in abundance under fertilised conditions (Ma *et al*., 2021; Chagnon *et al*., 2022), further supporting the idea that this family may not be most effective in supplying nutrients. Instead, this family may provide additional benefits, such as pathogen defence, which may be particularly beneficial under fertilised conditions (Sikes *et al*., 2009; Lekberg *et al*., 2021). The reduction in *Paraglomeraceae* abundance that we observed in our experiment in response to defoliation and increasing genotypic diversity may indicate that plants prioritised nutrient uptake in these conditions, while the absence of defoliation and monoculture conditions may have favoured associations with *Paraglomeraceae*, potentially due to higher pathogen pressure. It is worth noting that the ITS2 primers used for fungal amplification in this study do not target AM fungi specifically and can be sub‐optimal for detecting full changes in AM fungal communities (Suzuki *et al*., 2020). However, general fungal primers can still provide a robust overview of the AM fungal community, detect changes in relative abundance of some well‐detected families (Berruti *et al*., 2017; George *et al*., 2019) and capture similar shifts in community composition to environmental conditions (Lekberg *et al*., 2018). Further measurements of AM and pathogen root colonisation and the comparison of fungal community composition within roots and soil under field conditions are needed to test this hypothesis.

### Conclusions

Our study provides new mechanistic insights into how plants regulate soil microbial decomposer activity as an adaptive response to defoliation. We found that defoliation differentially enhanced ammonium rather than nitrate availability in soil and stimulated oxidative enzyme activities over others. This finding suggests that root exudation does not merely provide additional energy to microbial communities, but that shifts in exudate chemistry preferentially favour or suppress specific microbial functional groups. While many effects of defoliation and genotypic diversity were additive, some important interactive effects were identified. Defoliation enhanced root exudation and N mineralisation in both genotypic monocultures and mixtures, but genetically diverse plant groups secured a higher proportion of mineralised ^15^N in their leaves than monocultures showing the importance of plant genetic diversity in plant–microbial competition for N. This finding demonstrates how defoliation can interact with plant genetic diversity to affect N cycling and plant nutrition in grassland systems. The underlying mechanism requires further investigation. As defoliation and genotypic diversity increased root exudation and organic matter decomposition, while modifying decomposer community composition and decreasing incorporation of root exudates into microbial biomass, the changes in partitioning of mineralised N among plants and soil microbiome are likely driven by root exudate chemistry.

## Competing interests

None declared.

## Author contributions

MS conceived the idea. IC and MC contributed critically to designing methodology. IC, MC, ELF, AA, UK, HC, CH‐A, SW, SS and MS collected the data. IC, SIAF and MS analysed the data. MS led the writing of the first draft of the manuscript with critical inputs from IC. All authors contributed to the drafts and gave final approval for publication.

## Disclaimer

The New Phytologist Foundation remains neutral with regard to jurisdictional claims in maps and in any institutional affiliations.

## Supporting information


**Fig. S1** Effects of defoliation and genotypic diversity on the incorporation of ^13^C and ^15^N into microbial biomass.
**Fig. S2** Effects of defoliation and genotypic diversity on lignin oxidases in soil.
**Fig. S3** Relationships between root exudation and ^15^N atom% in plant leaves and soil enzymatic activity.
**Fig. S4** Principal coordinate analysis of fungal communities.
**Fig. S5** Effects of defoliation and genotypic diversity on the relative abundance of fungal pathogens.
**Fig. S6** Mean relative abundance of selected fungal families.
**Fig. S7** Mycorrhizal colonisation of roots.
**Table S1** Variation in plant traits in different genotypes.
**Table S2** Natural abundance of ^13^C and ^15^N isotopes.
**Table S3** Methodological details of enzyme analyses.
**Table S4** Effects of defoliation and genotypic diversity on photosynthesis, respiration and net ecosystem‐level carbon exchange.
**Table S5** Effects of defoliation and genotypic diversity on plant root and shoot biomass and traits.
**Table S6** Effects of defoliation and genotypic diversity on belowground carbon allocation.
**Table S7** Effects of defoliation and genotypic diversity on decomposer activity and nitrogen cycling.
**Table S8** Relationships between root exudation and either ^15^N incorporation into leaves or soil enzyme activity.
**Table S9** Effects of defoliation and genotypic diversity on fungal community composition.
**Table S10** Effects of defoliation and genotypic diversity on fungal species diversity and richness.
**Table S11** Most abundant putative plant pathogen ASVs.
**Table S12** Effects of defoliation and genotypic diversity on fungal relative abundance.
Please note: Wiley is not responsible for the content or functionality of any Supporting Information supplied by the authors. Any queries (other than missing material) should be directed to the *New Phytologist* Central Office.

## Data Availability

All data are deposited in Figshare repository under DOI: 10.6084/m9.figshare.32935700. Raw sequence reads are archived within the National Center for Biotechnology Information NCBI under project accession ID PRJNA1441981.
