# Supplementary material for: Plant genotypic diversity and defoliation jointly shape root exudation, fungal communities, and carbon and nitrogen cycling
Source: New Phytol. 2026 Aug 2;252(1):324–39. doi: 10.1111/nph.71479 (PMC13539967; doi:10.1111/nph.71479)
Supplement: Supplementary file 1 [file NPH-252-324-s001.pdf]

New Phytologist

***New Phytologist* Supporting Information**

Article title: Plant genotypic diversity and defoliation jointly shape root exudation, fungal communities and carbon and nitrogen cycling
Authors: Irene Cordero, Mathilde Chomel, Ellen L. Fry, Agnès Ardanuy, Ully Kritzler, Hayley Craig, Cristina Heredia-Acuña, Sarah Worthington, Stênio I. A. Foerster, Richard D. Bardgett, David Johnson, Marina Semchenko.
Article acceptance date: 15 July 2026

The following Supporting Information is available for this article:

Fig. S1 Effects of defoliation and genotypic diversity on the incorporation of ^{13}C and ^{15}N into microbial biomass.

Fig. S2 Effects of defoliation and genotypic diversity on lignin oxidases in soil.

Fig. S3 Relationships between root exudation and ^{15}N atom% in plant leaves and soil enzymatic activity.

Fig. S4 Principal coordinate analysis of fungal communities.

Fig. S5 Effects of defoliation and genotypic diversity on the relative abundance of fungal pathogens.

Fig. S6 Mean relative abundance of selected fungal families.

Fig. S7 Mycorrhizal colonisation of roots.

Table S1 Variation in plant traits in different genotypes.

Table S2 Natural abundance of ^{13}C and ^{15}N isotopes

Table S3 Methodological details of enzyme analyses.

Table S4 Effects of defoliation and genotypic diversity on photosynthesis, respiration and net ecosystem-level carbon exchange.

Table S5 Effects of defoliation and genotypic diversity on plant root and shoot biomass and traits.

Table S6 Effects of defoliation and genotypic diversity on belowground carbon allocation.

Table S7 Effects of defoliation and genotypic diversity on decomposer activity and nitrogen cycling.

Table S8 Relationships between root exudation and either ^{15}N incorporation into leaves or soil enzyme activity.

Table S9 Effects of defoliation and genotypic diversity on fungal community composition.

Table S10 Effects of defoliation and genotypic diversity on fungal species diversity and richness.

Table S11 Most abundant putative plant pathogen ASVs.

Table S12 Effects of defoliation and genotypic diversity on fungal relative abundance.

Fig. S1 Effects of defoliation (Unclipped or Clipped) and genotypic diversity (Monocultures or Mixtures) of *Anthoxanthum odoratum* on the incorporation of (a) ^{13}C into microbial biomass (in µg per g of dry soil) relative to ^{13}C exuded (in µg) and (b) ^{15}N into microbial biomass (in µg per g of dry soil) relative to ^{15}N atom% in plant leaves. Solid-colored dots represent the average predictions from linear mixed models, while whiskers indicate the 95% confidence intervals for these predictions. Statistical significance is indicated in the panel labels for model terms: genotypic diversity (M), grazing (C), and their interaction (MxC). *P*-value codes: * $P < 0.05$, ns $P > 0.05$.

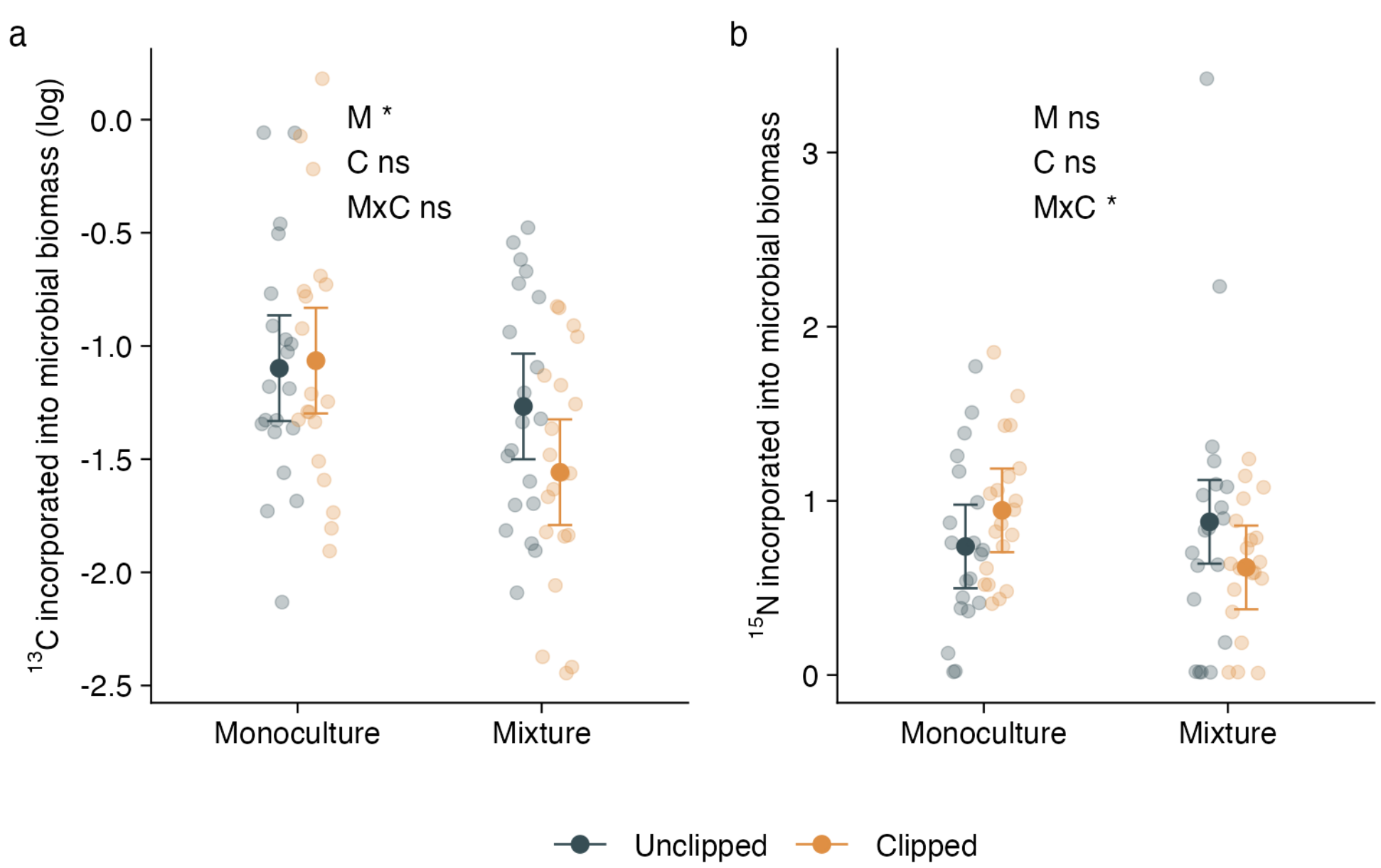

Fig. S2 Effects of defoliation (Unclipped or Clipped) and genotypic diversity (Monocultures or Mixtures) of *Anthoxanthum odoratum* on lignin oxidases in soil. Solid-colored dots represent the average predictions from linear mixed models, while whiskers indicate the 95% confidence intervals for these predictions. Statistical significance is indicated in the panel labels for model terms: grazing (C), genotypic diversity (M), and the interaction between grazing and genotypic diversity (MxC).

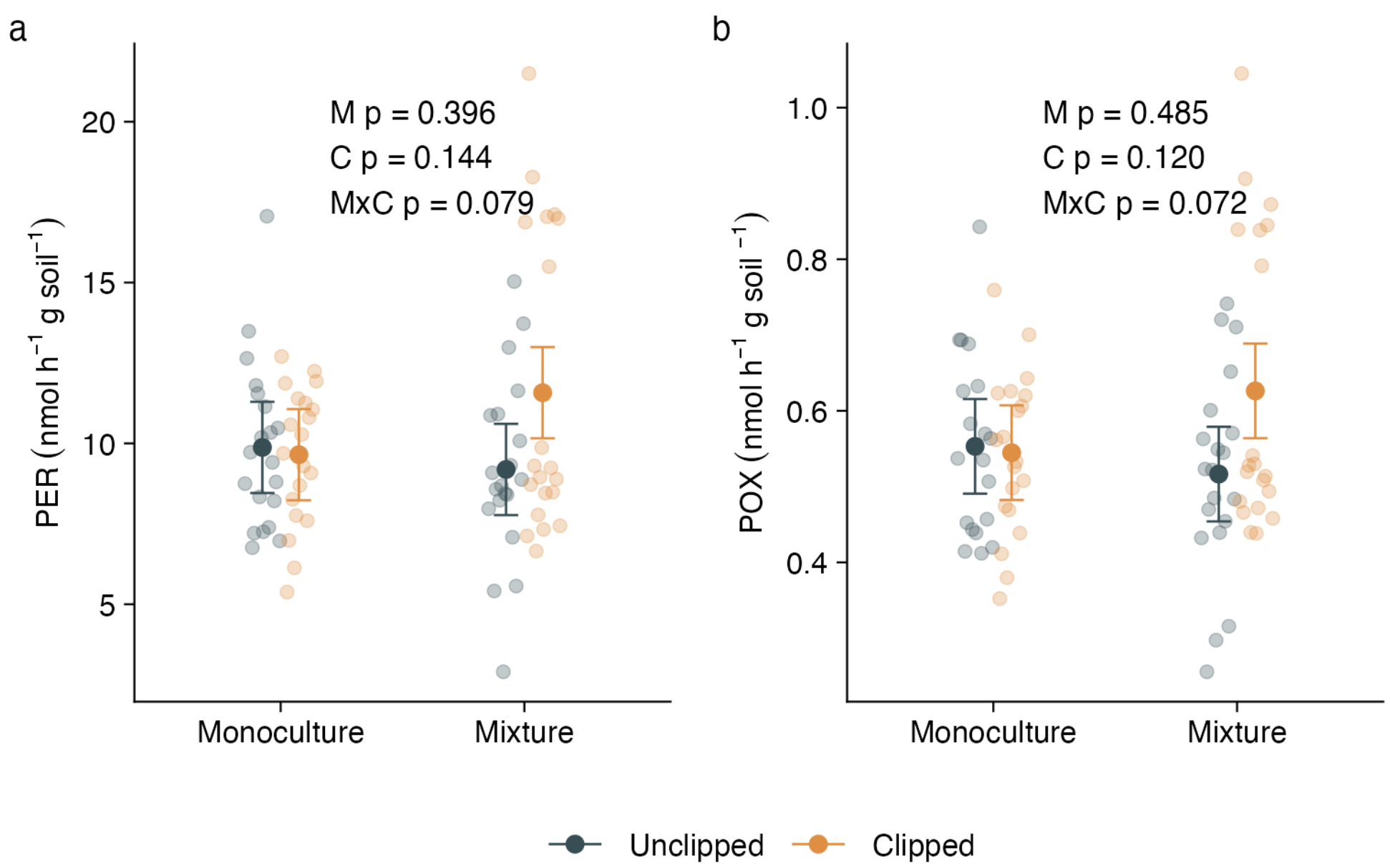

Fig. S3 Relationships between root exudation (measured as ^{13}C in soil leachates after pulse labelling) and ^{15}N atom% in plant leaves of *Anthoxanthum odoratum* and soil enzymatic activity in unclipped (in grey) and defoliation (clipped, in orange) treatments. Shaded areas show the 95% confidence intervals around the prediction lines obtained from mixed linear models. PER – peroxidase. POX –phenol oxidase. See Table S8 for statistics.

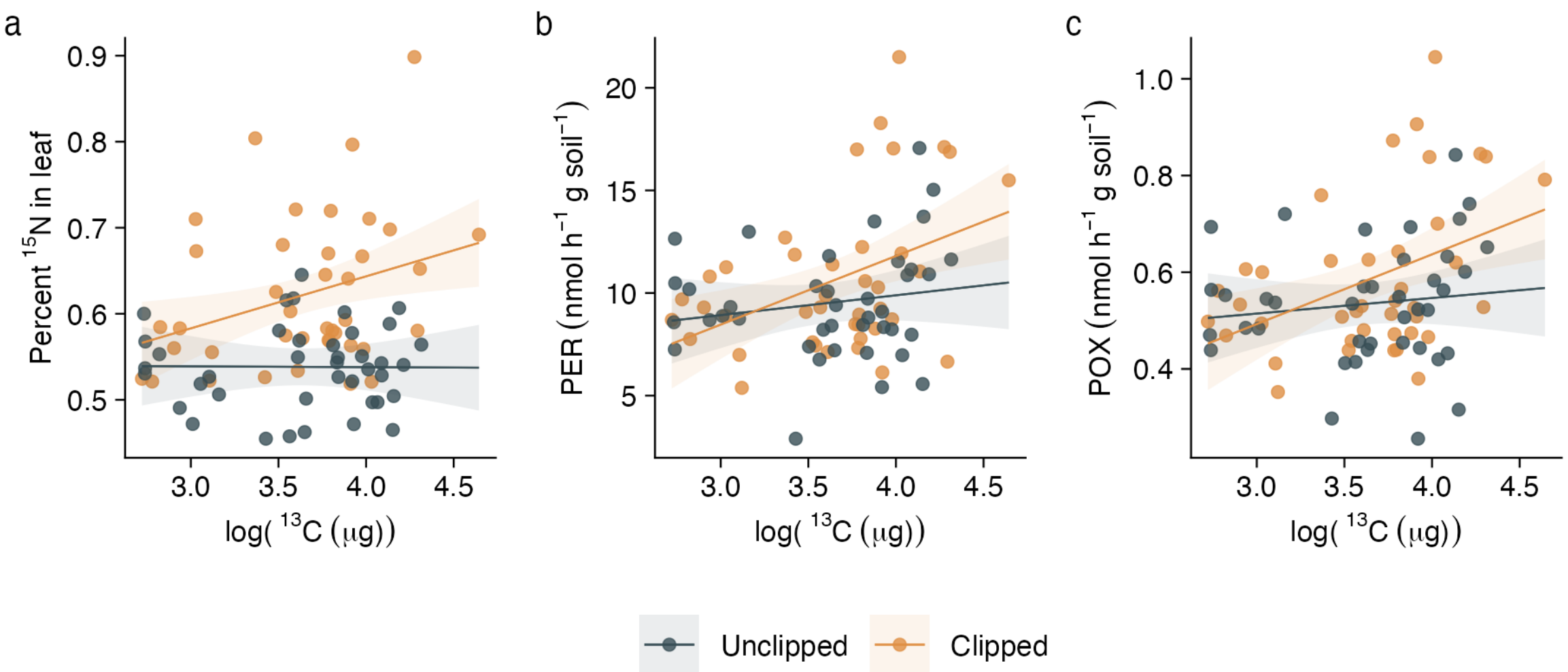

Fig. S4 Principal coordinate analysis of a) whole soil fungal communities, b) putative pathogen fungal communities, c) arbuscular mycorrhizal fungal communities and d) saprotroph fungal communities associated with *Anthoxanthum odoratum*. Centroids and standard errors are shown for four defoliation and genotypic diversity treatment combinations. For all four analyses, the effect of clipping was significant at $P < 0.05$ (see Table S9 for detailed results of PERMANOVA analysis).

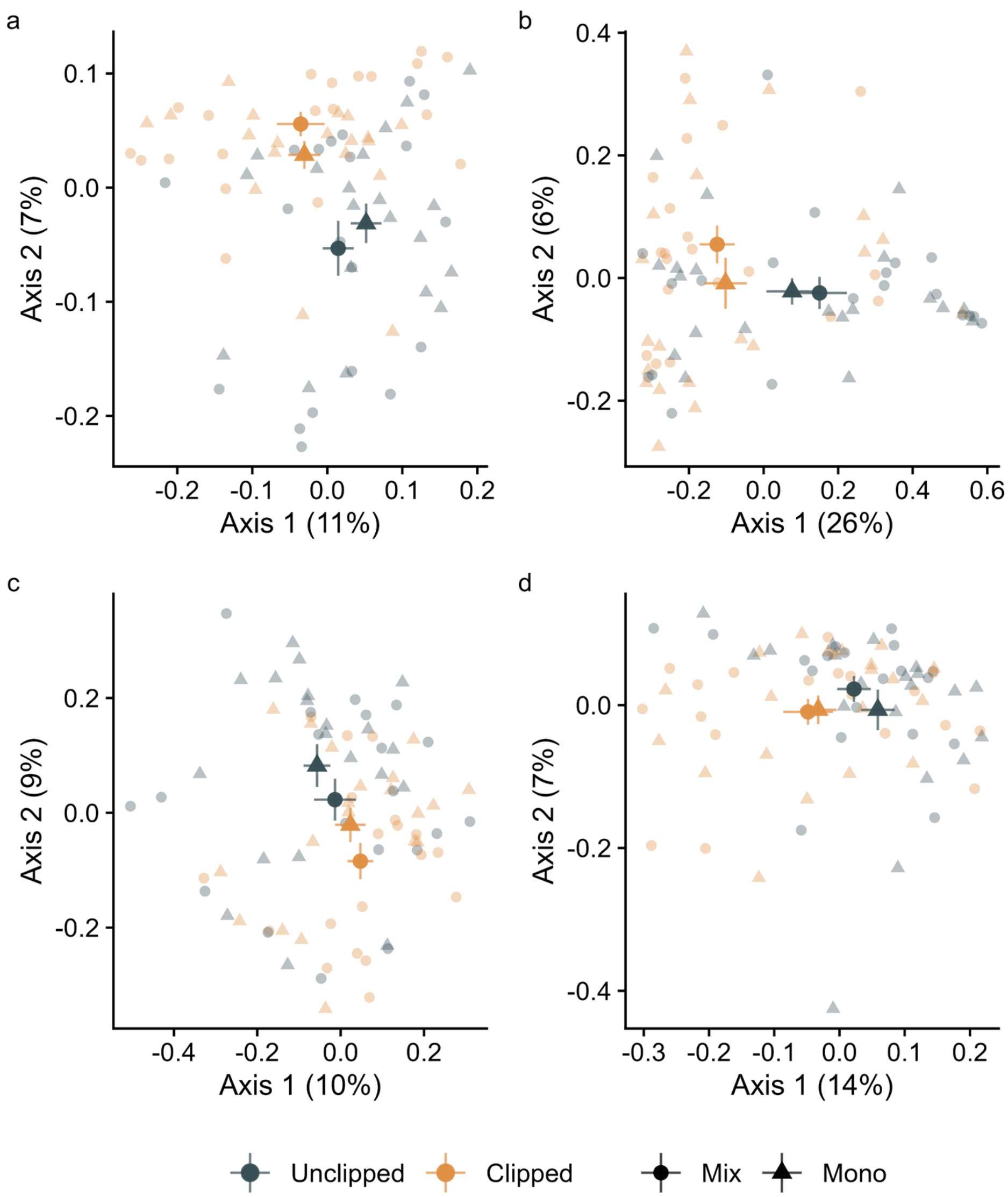

Fig. S5 Effects of defoliation (Unclipped or Clipped) and genotypic diversity (Monocultures or Mixtures) on the relative abundance of fungal pathogens in association with *Anthoxanthum odoratum*. The analyses were conducted (a) with and (b) without including the numerically dominant OTU ASV11. Solid-colored dots represent the average predictions from linear mixed models, while whiskers indicate the 95% confidence intervals for these predictions. Statistical significance is indicated in the panel labels for model terms: simulated grazing by clipping (C), genotypic diversity (M), sequence depth (S), and the interaction between grazing and genotypic diversity (MxC). P-value codes: ns $P > 0.05$, * $P < 0.05$, ** $P < 0.01$, *** $P < 0.001$.

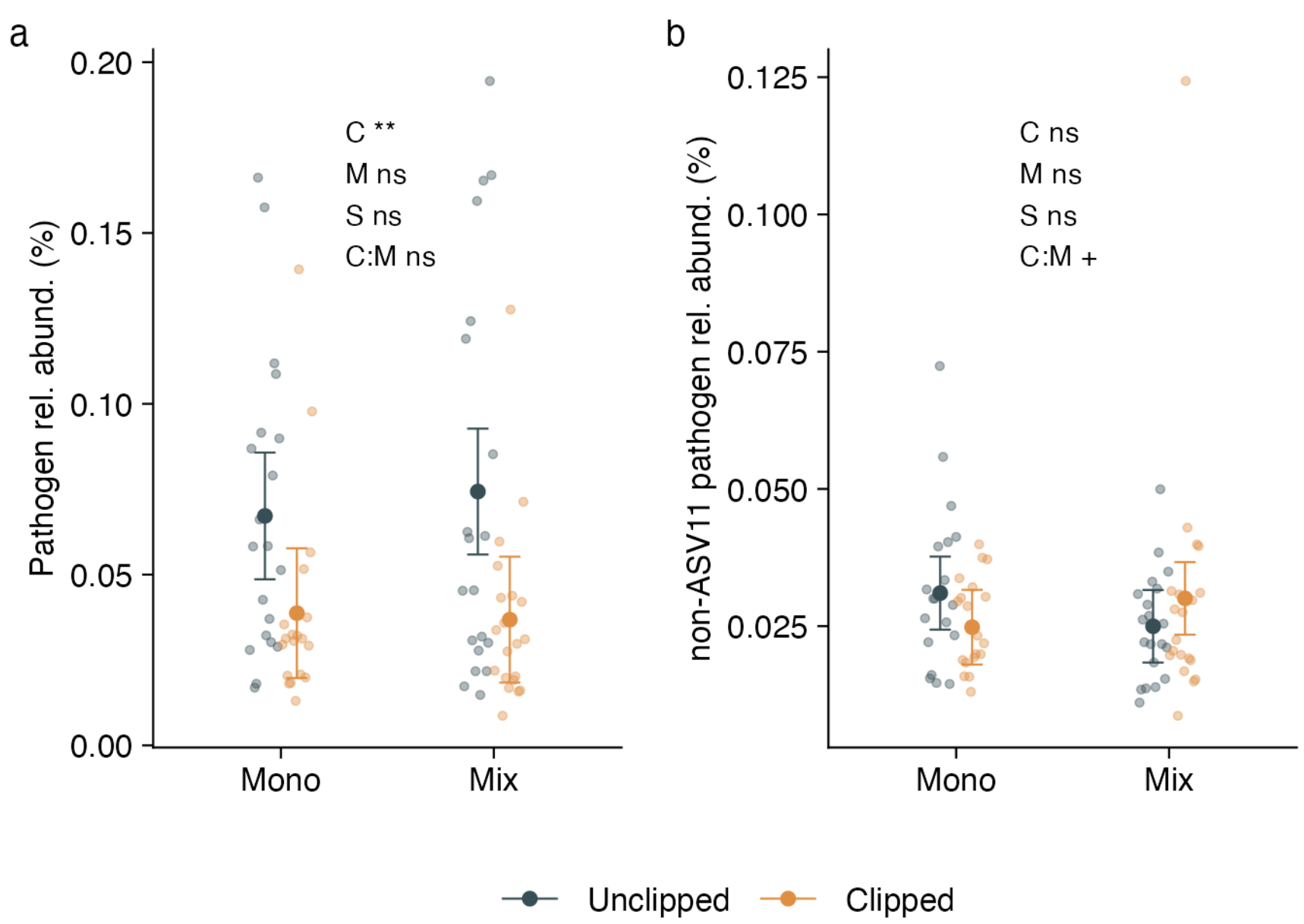

Fig. S6 Mean relative abundance of fungal families in genotypic monocultures (Mono) and mixtures (Mix) of *Anthoxanthum odoratum* in unclipped and clipped treatment. The significance of model terms explaining variation in the relative abundance of each fungal group is shown as follows: M – effect of genotypic diversity, C – effect of clipping; ns $P > 0.1$, + $P < 0.1$, * $P < 0.05$, ** $P < 0.01$, *** $P < 0.001$.

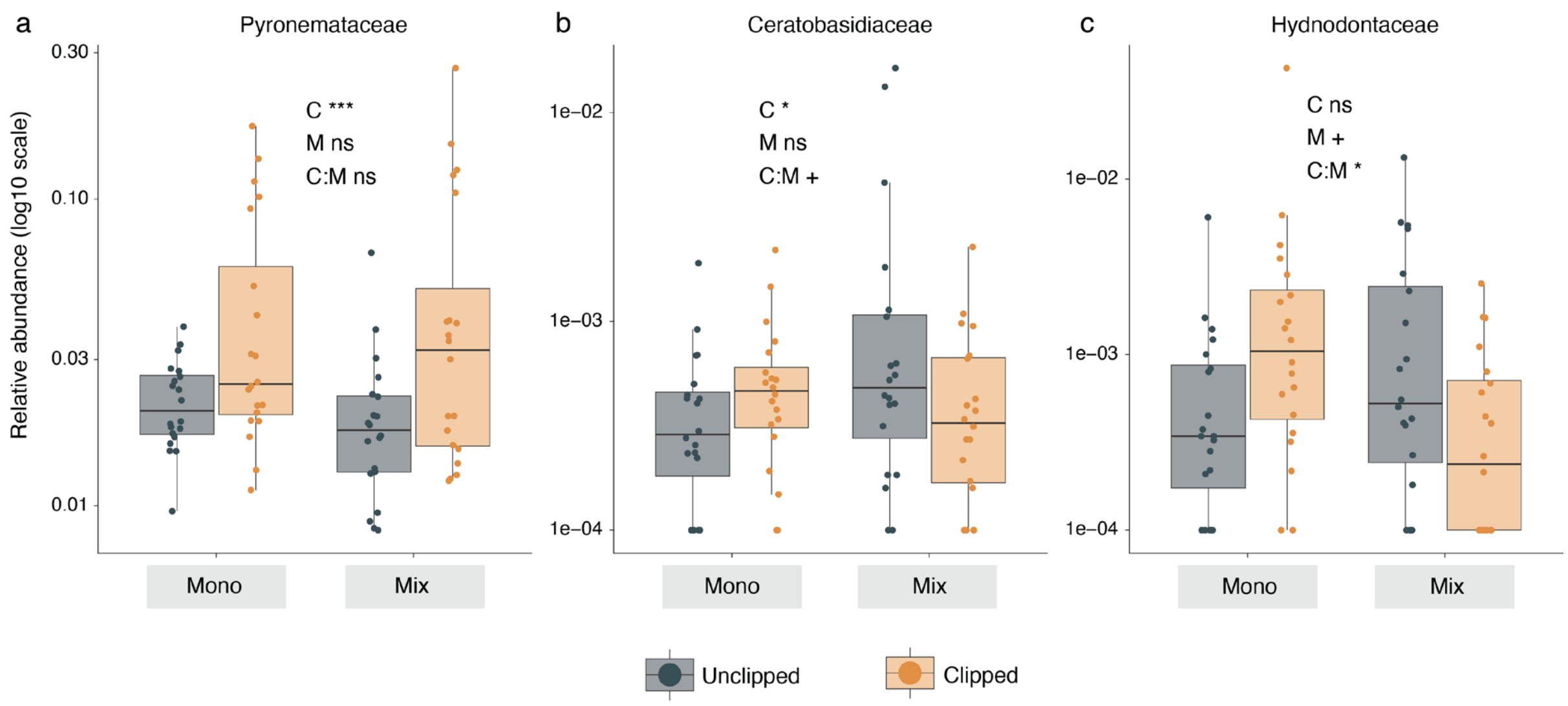

Fig. S7 Mycorrhizal fungi colonisation in a subset of root samples of *Anthoxanthum odoratum* from genotypic monocultures (Mono) and mixtures (Mix) in unclipped and clipped treatment. There was no significant effect: clipping: $P = 0.51$, genotypic diversity: $P = 0.11$, their interaction: $P = 0.21$. n = 4-5.

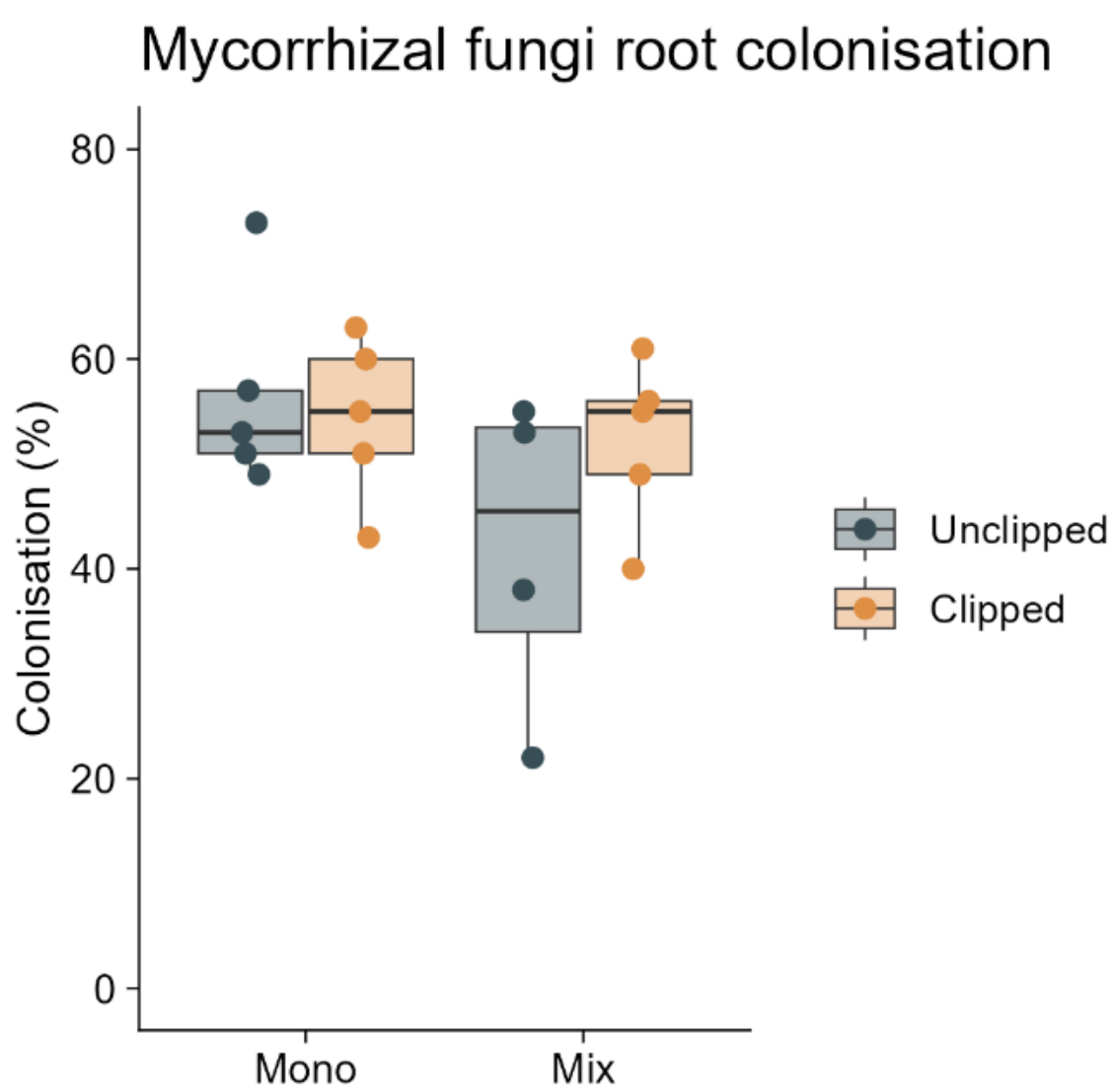

Table S1 Variation in plant traits in different genotypes of Anthoxanthum odoratum. Values from monoculture and unclipped plants. n = 20. CV = coefficient of variation. Trait variation refers to how many times bigger the maximum value is with respect to the minimum; maximum value / minimum value.

Variable	Mean	Median	Minimum value	Maximum value	Standard deviation	CV	Trait variation
Total shoot dry mass (g)	3.80	3.68	2.62	5.24	0.70	0.18	2.00
Total root dry mass (g)	5.54	5.49	3.29	10.38	1.67	0.30	3.16
Total dry mass (g)	9.33	9.30	5.91	14.16	2.02	0.22	2.40
Root to shoot mass ratio	1.47	1.39	0.84	2.75	0.42	0.28	3.28
Mean plant height before clipping (cm)	8.29	8.40	5.90	11.20	1.42	0.17	1.90
Mean plant height at harvest (cm)	9.18	9.23	6.80	11.60	1.57	0.17	1.71
Specific leaf area (cm^2 mg^{-1})	0.24	0.23	0.18	0.30	0.03	0.12	1.69
Leaf dry matter content	0.30	0.30	0.26	0.35	0.02	0.07	1.35
Leaf nitrogen (%)	1.35	1.40	0.93	1.81	0.25	0.18	1.95
Leaf carbon to nitrogen ratio	33.66	31.66	24.24	45.56	6.42	0.19	1.88
Root diameter (mm)	0.15	0.15	0.13	0.17	0.01	0.07	1.31
Specific root length (cm mg^{-1})	39.95	39.97	25.96	52.36	6.40	0.16	2.02
Root tissue density (g cm^3)	0.15	0.15	0.13	0.19	0.01	0.10	1.50
Root nitrogen (%)	0.45	0.44	0.38	0.54	0.04	0.10	1.42
Root carbon to nitrogen ratio	97.30	98.27	79.96	114.69	10.31	0.11	1.43
Soil microbial biomass C (mg · g^{-1} dry soil)	1.09	1.14	0.53	1.62	0.26	0.24	3.07

Table S2 Natural abundance of ^{13}C and ^{15}N isotopes in unlabelled samples and the range of enrichment in labelled samples.

	Natural abundance	Min. enriched	Max. enriched
$\delta^{13}C$ in leaves clipped at the end of labelling	-30.07	150.12	341.8
$\delta^{13}C$ in soil leachates	-30.83	-27.87	9.71
$\delta^{13}C$ in soil water 48h after labelling	-32.28	-40.44	-3.39
$\delta^{13}C$ in fumigated soil samples	-27.63	-26.97	1.09
$\delta^{15}N$ in leaves	9.26	242.65	1465.82
$\delta^{15}N$ in fumigated soil samples	13.15	38.28	1230.54

Table S3 Methodological details of enzyme analyses. All enzymes were incubated at 18 °C under continuous shaking.

Enzyme	Full name	Substrate	Incubation time	[Substrate]
GLC	β-glucosidase	pNP-β-glucopyranoside	1.5 h	30 mM
CBH	Cellobiohydrolase	pNP-β-D-cellobioside	4 h	2 mM
XYL	Xylosidase	pNP-β-xylopyranoside	3.5 h	30 mM
NAG	N- acetylglucosaminidase	pNP-N-acetyl-β-D-glucosaminide	3.5 h	8 mM
AP	Acid phosphatase	pNP-phosphate disodium salt hexahydrate	30 min	40 mM
URE	Urease	Urea	2 h	100 mM
POX	Phenol oxidase	L-3,4-dihydroxyphenylalanin (L-DOPA)	18 h	20 mM
PER	Peroxidase	L-3,4-dihydroxyphenylalanin (L-DOPA) + H_2O_2	1.5 h	20 mM

Table S4 Effects of defoliation (Clipping) and genotypic diversity (Mix) on photosynthesis, respiration and net ecosystem-level carbon exchange in the temperate grass *Anthoxanthum odoratum* across a 10-week greenhouse experiment. The effects were estimated using linear mixed models accounting for the genotypic composition of the samples (intercept-level random factor) and including mean light availability (Mean_PAR) as a covariate. Degrees of freedom are reported for fixed effects (Df) and model residuals (Df.res). F- and P- values are shown; P-values lower than 0.1 are shown in italic and lower than 0.05 in bold.

**Response**	**Time**	**Term**	**F**	**Df**	**Df.res**	***P***
Photosynthesis	Before clipping	Mix	0.12	1	37.37	0.730
		Clipping	1.89	1	37.52	0.177
		Mean_PAR	10.20	1	71.46	**0.002**
		Mix*Clipping	4.07	1	40.57	*0.050*
	24 Hours	Mix	1.08	1	37.18	0.306
		Clipping	303.41	1	38.15	**< 0.001**
		Mean_PAR	1.85	1	40.99	0.181
		Mix*Clipping	0.05	1	38.05	0.832
	2 Weeks	Mix	4.28	1	37.41	**0.046**
		Clipping	6.95	1	37.16	**0.012**
		Mean_PAR	2.44	1	70.65	0.122
		Mix*Clipping	0.003	1	39.21	0.959
	10 Weeks	Mix	0.0003	1	35.54	0.987
		Clipping	1.15	1	36.59	0.290
		Mean_PAR	59.57	1	40.22	**< 0.001**
		Mix*Clipping	0.01	1	36.56	0.933
Respiration	Before clipping	Mix	0.49	1	37.35	0.486
		Clipping	1.09	1	37.54	0.303
		Mean_PAR	5.73	1	72.63	**0.019**
		Mix*Clipping	4.24	1	40.66	**0.046**
	24 Hours	Mix	0.18	1	37.28	0.671
		Clipping	17.22	1	38.23	**< 0.001**
		Mean_PAR	34.05	1	43.65	**< 0.001**
		Mix*Clipping	0.03	1	38.07	0.864
	2 Weeks	Mix	8.37	1	37.41	**0.006**
		Clipping	29.65	1	37.16	**< 0.001**
		Mean_PAR	0.05	1	70.65	0.831
		Mix*Clipping	1.01	1	39.21	0.321
	10 Weeks	Mix	0.41	1	35.54	0.526
		Clipping	12.65	1	36.59	**0.001**
		Mean_PAR	0.17	1	40.22	0.684
		Mix*Clipping	0.14	1	36.56	0.715

Response	Time	Term	F	Df	Df.res	*P*
Net ecosystem exchange	Before clipping	Mix	0.69	1	37.46	0.412
		Clipping	0.93	1	37.42	0.341
		Mean_PAR	3.74	1	66.09	*0.058*
		Mix*Clipping	0.81	1	40.16	0.375
	24 Hours	Mix	0.31	1	37.23	0.582
		Clipping	220.54	1	38.19	< **0.001**
		Mean_PAR	24.07	1	42.30	< **0.001**
		Mix*Clipping	0.004	1	38.06	0.951
	2 Weeks	Mix	0.05	1	37.41	0.817
		Clipping	37.09	1	37.16	< **0.001**
		Mean_PAR	2.89	1	70.65	*0.094*
		Mix*Clipping	0.34	1	39.21	0.562
	10 Weeks	Mix	0.22	1	36.75	0.645
		Clipping	2.49	1	36.70	0.123
		Mean_PAR	66.49	1	44.22	< **0.001**
		Mix*Clipping	0.00001	1	36.57	0.997

Table S5 Effects of defoliation (Clipping) and genotypic diversity (Mix) on plant root and shoot biomass and traits in the temperate grass *Anthoxanthum odoratum* over a 10-week greenhouse experiment. Effects were estimated using linear mixed models, with the genotypic composition of the samples included as an intercept-level random factor. Response variables were log-transformed (natural logarithms) to satisfy model assumptions. Degrees of freedom for fixed effects and model residuals were invariant (1 and 38, respectively) across all models and are therefore omitted from the table. F- and P- values are shown; P-values lower than 0.1 are shown in italic and lower than 0.05 in bold.

**Response**	**Term**	**F**	***P***
Total shoot dry mass (log g)	Mix	1.64	0.208
	Clipping	1.37	0.249
	Mix*Clipping	0.79	0.379
Total root dry mass (log g)	Mix	0.10	0.759
	Clipping	7.20	**0.011**
	Mix*Clipping	0.07	0.791
Total dry mass (log g)	Mix	0.53	0.472
	Clipping	3.18	*0.083*
	Mix*Clipping	0.16	0.690
Root to shoot mass ratio (log)	Mix	0.26	0.611
	Clipping	10.49	**0.002**
	Mix*Clipping	0.04	0.841
Mean plant height before clipping (log cm)	Mix	0.002	0.961
	Clipping	0.68	0.414
	Mix*Clipping	0.03	0.873
Coefficient of variation in plant height before clipping (log)	Mix	10.71	**0.002**
	Clipping	0.14	0.713
	Mix*Clipping	0.03	0.874
Mean plant height at harvest (log cm)	Mix	0.002	0.963
	Clipping	69.74	**< 0.001**
	Mix*Clipping	0.74	0.395
Coefficient of variation in plant height at harvest (log)	Mix	22.76	**< 0.001**
	Clipping	0.18	0.673
	Mix*Clipping	0.19	0.664
Specific leaf area (log cm^2 mg^{-1})	Mix	0.11	0.745
	Clipping	2.01	0.165
	Mix*Clipping	0.21	0.650
Leaf dry matter content (log)	Mix	0.01	0.934
	Clipping	0.82	0.370
	Mix*Clipping	0.55	0.465

**Response**	**Term**	**F**	***P***
Leaf nitrogen (log %)	Mix	1.50	0.228
	Clipping	4.34	**0.044**
	Mix*Clipping	0.11	0.738
Leaf carbon to nitrogen ratio (log)	Mix	1.54	0.222
	Clipping	5.15	**0.029**
	Mix*Clipping	0.19	0.668
Root diameter (log mm)	Mix	1.19	0.282
	Clipping	2.29	0.138
	Mix*Clipping	0.28	0.599
Specific root length (log cm mg^{-1})	Mix	0.10	0.750
	Clipping	0.17	0.684
	Mix*Clipping	1.38	0.247
Root tissue density (log g cm^{3})	Mix	1.25	0.270
	Clipping	4.08	0.*050*
	Mix*Clipping	0.39	0.538
Root nitrogen (log %)	Mix	0.53	0.469
	Clipping	1.16	0.289
	Mix*Clipping	0.31	0.583
Root carbon to nitrogen ratio (log)	Mix	0.79	0.381
	Clipping	0.90	0.348
	Mix*Clipping	0.69	0.412

Table S6 Effects of defoliation (Clipping) and genotypic diversity (Mix) on belowground carbon allocation including root exudation in the temperate grass *Anthoxanthum odoratum* over a 10-week greenhouse experiment. Effects were estimated using linear mixed models, with the genotypic composition of the samples included as an intercept-level random factor. Response variables were log-transformed (natural logarithms) to satisfy model assumptions. Degrees of freedom for fixed effects and model residuals were invariant (1 and 38, respectively) across all models and are therefore omitted from the table. F- and P- values are shown; P-values lower than 0.1 are shown in italic and lower than 0.05 in bold.

**Response**	**Term**	**F**	***P***
Soil leachate ^{13}C, 2h after labelling (log atom%)	Mix	2.60	0.115
	Clipping	0.51	0.480
	Mix*Clipping	0.12	0.731
Soil leachate ^{13}C, 2h after labelling (log µg)	Mix	7.84	**0.008**
	Clipping	0.02	0.889
	Mix*Clipping	2.64	0.113
Dissolved soil organic ^{13}C, 48 h after labelling (log µg · g^{-1} dry soil)	Mix	6.21	**0.017**
	Clipping	0.02	0.880
	Mix*Clipping	3.37	*0.074*
Dissolved soil organic C, 48h after labelling (log mg · g^{-1} dry soil)	Mix	6.03	**0.019**
	Clipping	0.45	0.506
	Mix*Clipping	1.89	0.177
Microbial biomass ^{13}C (log µg · g^{-1} dry soil)	Mix	0.12	0.728
	Clipping	4.23	**0.047**
	Mix*Clipping	0.09	0.772
Microbial biomass C (log mg · g^{-1} dry soil)	Mix	0.13	0.721
	Clipping	3.38	*0.074*
	Mix*Clipping	0.07	0.792
Root exudation before clipping (mg C)	Mix	0.19	0.668
	Clipping	1.91	0.175
	Mix*Clipping	2.02	0.164
Root exudation 3 days after clipping (mg C)	Mix	0.53	0.472
	Clipping	9.55	**0.004**
	Mix*Clipping	1.42	0.240
Soil leachate organic C, 2h after labelling (mg)	Mix	7.81	**0.008**
	Clipping	0.0001	0.991
	Mix*Clipping	2.77	0.104
^{13}C incorporated into microbial biomass relative to ^{13}C exuded	Mix	6.44	**0.015**
	Clipping	1.45	0.235
	Mix*Clipping	2.31	0.137

Table S7 Effects of defoliation (Clipping) and genotypic diversity (Mix) on decomposer activity and nitrogen cycling in the temperate grass *Anthoxanthum odoratum* over a 10-week greenhouse experiment. Effects were estimated using linear mixed models, with the genotypic composition of the samples included as an intercept-level random factor. Degrees of freedom for fixed effects and model residuals were invariant (1 and 38, respectively) across all models and are therefore omitted from the table. GLC – β-glucosidase, XYL – xylosidase, CBH – cellobiohydrolase, NAG – N- acetylglucosaminidase, AP – acid phosphatase, URE– urease, PER – peroxidase and POX – phenoloxidase). F- and P- values are shown; P-values lower than 0.1 are shown in italic and lower than 0.05 in bold.

**Response**	**Term**	**F**	***P***
Proportion of leaf litter decomposed	Mix	0.47	0.498
	Clipping	4.93	**0.032**
	Mix*Clipping	0.02	0.883
Proportion of root litter decomposed	Mix	0.14	0.711
	Clipping	6.01	**0.019**
	Mix*Clipping	0.05	0.825
Ammonium availability in soil (μg · g^{-1} dry soil)	Mix	0.46	0.502
	Clipping	12.47	**0.001**
	Mix*Clipping	2.84	0.100
Nitrate availability in soil (μg · g^{-1} dry soil)	Mix	1.82	0.185
	Clipping	1.22	0.276
	Mix*Clipping	0.86	0.360
GLC (nmol · h^{-1} · g^{-1} soil)	Mix	0.47	0.495
	Clipping	0.05	0.819
	Mix*Clipping	1.09	0.304
XYL (nmol · h^{-1} · g^{-1} soil)	Mix	0.45	0.505
	Clipping	0.17	0.683
	Mix*Clipping	0.14	0.709
CBH (nmol · h^{-1} · g^{-1} soil)	Mix	0.05	0.821
	Clipping	0.16	0.695
	Mix*Clipping	0.67	0.417
NAG (nmol · h^{-1} · g^{-1} soil)	Mix	0.75	0.391
	Clipping	0.33	0.571
	Mix*Clipping	0.49	0.486
AP (nmol · h^{-1} · g^{-1} soil)	Mix	0.65	0.425
	Clipping	0.24	0.626
	Mix*Clipping	4.62	**0.038**
URE (μg NH_4+· h^{-1} · g^{-1} soil)	Mix	3.13	*0.085*
	Clipping	0.01	0.905

Response	Term	F	*P*
	Mix*Clipping	0.05	0.825
PER (nmol · h^{-1} · g^{-1} soil)	Mix	0.74	0.396
	Clipping	2.23	0.144
	Mix*Clipping	3.26	*0.079*
POX (nmol · h^{-1} · g^{-1} soil)	Mix	0.50	0.485
	Clipping	2.54	0.120
	Mix*Clipping	3.43	*0.072*
Leaf ^{15}N (%)	Mix	0.47	0.498
	Clipping	33.48	**< 0.001**
	Mix*Clipping	1.61	0.213
Root ^{15}N (%)	Mix	0.16	0.695
	Clipping	18.45	**< 0.001**
	Mix*Clipping	2.53	0.120
Total ^{15}N in leaves (µg)	Mix	0.0002	0.990
	Clipping	6.27	**0.017**
	Mix*Clipping	0.02	0.887
Total ^{15}N in roots (µg)	Mix	0.01	0.907
	Clipping	3.46	*0.071*
	Mix*Clipping	0.005	0.946
^{15}N in soil solution (%)	Mix	1.66	0.206
	Clipping	2.09	0.157
	Mix*Clipping	7.87	**0.008**
Microbial biomass N (mg · g-1 dry soil)	Mix	2.30	0.138
	Clipping	1.69	0.202
	Mix*Clipping	4.19	**0.048**
Microbial biomass ^{15}N (µg · g-1 dry soil)	Mix	1.16	0.288
	Clipping	0.94	0.339
	Mix*Clipping	4.75	**0.036**
^{15}N incorporated into microbial biomass relative to plant leaves (%)	Mix	0.45	0.506
	Clipping	0.06	0.812
	Mix*Clipping	5.02	**0.031**

Table S8 Relationships between root exudation (assessed as the amount of ^{13}C in soil leachate 2 hours after pulse labeling) and either ^{15}N incorporation into leaves from decomposing litter or soil enzyme activity in the temperate grass *Anthoxanthum odoratum*. Effects were estimated using linear mixed models, with exudation (in µg of ^{13}C, ln-transformed) included as a fixed factor and the genotypic composition of the samples included as an intercept-level random factor, which was removed in case of singular fit. Degrees of freedom are reported for fixed effects (Df) and model residuals (Df.res). GLC – β-glucosidase, XYL – xylosidase, CBH – cellobiohydrolase, NAG – N- acetylglucosaminidase, AP – acid phosphatase, URE– urease, PER – peroxidase and POX – phenoloxidase. F- and P- values are shown; P-values lower than 0.1 are shown in italic and lower than 0.05 in bold.

**Response**	**Term**	**F**	**Df**	**Df.res**	**Pr(>F)**
Leaf ^{15}N (atom%)	^{13}C	2.78	1	75.4	*0.0995*
	Clipping	34.82	1	37.4	**<0.001**
	^{13}C*Clipping	3.52	1	65.3	*0.065*
GLC (nmol · h^{-1} · g^{-1} soil)	^{13}C	1.73	1	73.0	0.193
	Clipping	0.06	1	37.2	0.802
	^{13}C*Clipping	0.03	1	54.7	0.865
XYL (nmol · h^{-1} · g^{-1} soil)	^{13}C	1.68	1	75.3	0.199
	Clipping	0.16	1	37.3	0.694
	^{13}C*Clipping	0.00	1	58.3	0.979
CBH (nmol · h^{-1} · g^{-1} soil)	^{13}C	0.19	1	71.4	0.660
	Clipping	0.16	1	37.5	0.690
	^{13}C*Clipping	0.67	1	71.4	0.417
NAG (nmol · h^{-1} · g^{-1} soil)	^{13}C	1.00	1	71.4	0.320
	Clipping	0.35	1	37.5	0.560
	^{13}C*Clipping	0.97	1	71.4	0.328
AP (nmol · h^{-1} · g^{-1} soil)	^{13}C	0.07	1	75.6	0.786
	Clipping	0.21	1	37.4	0.651
	^{13}C*Clipping	0.44	1	64.7	0.511
URE (µg NH_4^+ · h^{-1} · g^{-1} soil)	^{13}C	1.11	1	71.7	0.297
	Clipping	0.01	1	37.5	0.918
	^{13}C*Clipping	0.06	1	71.2	0.805
PER (nmol · h^{-1} · g^{-1} soil)	^{13}C	7.50	1	72.6	**0.008**
	Clipping	2.37	1	37.5	0.132
	^{13}C*Clipping	2.43	1	70.2	0.123

POX (nmol · h^{-1} · g^{-1} soil)	^{13}C	6.43	1	71.9	**0.013**
	Clipping	2.64	1	37.5	0.113
	^{13}C*Clipping	2.74	1	70.9	0.102

Table S9 Effects of defoliation (Clipping) and genotypic diversity (Mix) of *Anthoxanthum odoratum* on fungal community composition, estimated using permutational analysis of variance (PERMANOVA). The analysis was conducted for all fungi as well as for functional groups of taxonomic operational units. Statistical significance was assessed using 1000 permutations. Abbreviations: Df = degrees of freedom, SS = sum of squares, R^2 = coefficient of determination. AM – arbuscular mycorrhizal.). P-values lower than 0.1 are shown in italic and lower than 0.05 in bold.

Response	Term	Df	SS	R^2	F	*P*
All fungi	Clipping	1	0.32	0.04	3.05	**<0.001**
	Mix	1	0.11	0.01	1.05	0.359
	Clipping*Mix	1	0.10	0.01	0.96	0.513
	Residual	76	8.02	0.94		
	Total	79	8.55	1.00		
Pathogens	Clipping	1	1.34	0.05	3.93	**<0.001**
	Mix	1	0.27	0.01	0.79	0.681
	Clipping*Mix	1	0.34	0.01	1.00	0.339
	Residual	76	25.89	0.93		
	Total	79	27.84	1.00		
Pathogens without ASV11	Clipping	1	0.25	0.02	1.31	0.119
	Mix	1	0.16	0.01	0.83	0.746
	Clipping*Mix	1	0.17	0.01	0.85	0.705
	Residual	76	14.71	0.96		
	Total	79	15.29	1.00		
AM Fungi	Clipping	1	0.58	0.02	1.96	**<0.001**
	Mix	1	0.34	0.01	1.15	0.200
	Clipping*Mix	1	0.30	0.01	1.02	0.390
	Residual	76	22.36	0.95		
	Total	79	23.57	1.00		
Saprotrophs	Clipping	1	0.32	0.03	2.45	**0.002**
	Mix	1	0.14	0.01	1.07	0.319
	Clipping*Mix	1	0.11	0.01	0.82	0.783
	Residual	76	10.03	0.95		
	Total	79	10.60	1.00		

Table S10 Effects of defoliation (Clipping) and genotypic diversity (Mix) of *Anthoxanthum odoratum* on fungal species diversity (Shannon index) and richness estimated using linear mixed models. Separate models were fitted for all fungi as well as for functional subgroups. The plant genotypic composition of the pot was included as an intercept-level random factor, and the sequence depth as a covariate to account for potential differences in sequencing effort. Degrees of freedom are reported for fixed effects (Df) and model residuals (Df.res). F- and P-values are shown; P-values lower than 0.1 are shown in italic and lower than 0.05 in bold.

		Diversity				Richness			
**Response**	**Term**	**F**	**Df**	**Df.res**	***P***	**F**	**Df**	**Df.res**	***P***
All fungi	Clipping	0.11	1	37.1	0.740	0.63	1	36.9	0.432
	Mix	**4.89**	**1**	**37.0**	**0.033**	**8.73**	**1**	**37.2**	**0.005**
	log(seq_depth)	*3.64*	*1*	*72.0*	*0.060*	**63.12**	**1**	**73.9**	**<0.001**
	Clipping*Mix	0.01	1	37.1	0.931	2.18	1	36.9	0.148
Pathogens	Clipping	**12.33**	**1**	**37.1**	**0.001**	0.01	1	37.0	0.940
	Mix	0.16	1	37.0	0.689	2.04	1	37.1	0.162
	log(seq_depth)	0.36	1	72.0	0.553	**9.64**	**1**	**73.3**	**0.003**
	Clipping*Mix	0.84	1	37.1	0.364	0.09	1	37.0	0.764
Pathogens without ASV11	Clipping	*3.14*	*1*	*36.7*	*0.085*	0.08	1	37.0	0.775
	Mix	0.03	1	37.5	0.865	1.83	1	37.1	0.185
	log(seq_depth)	**6.20**	**1**	**72.6**	**0.015**	**10.50**	**1**	**73.3**	**0.002**
	Clipping*Mix	0.10	1	36.7	0.750	0.13	1	37.0	0.725
AM fungi	Clipping	0.39	1	36.9	0.534	0.03	1	37.1	0.864
	Mix	0.85	1	37.3	0.364	0.79	1	37.0	0.381
	log(seq_depth)	**13.81**	**1**	**74.0**	**<0.001**	**23.77**	**1**	**72.0**	**<0.001**
	Clipping*Mix	0.01	1	36.9	0.924	0.40	1	37.1	0.532
Saprotrophs	Clipping	0.67	1	37.0	0.418	0.22	1	37.0	0.641
	Mix	2.70	1	37.1	0.109	**5.99**	**1**	**37.1**	**0.019**
	log(seq_depth)	**4.69**	**1**	**73.4**	**0.034**	**44.93**	**1**	**73.3**	**<0.001**
	Clipping*Mix	0.03	1	37.0	0.871	0.06	1	37.0	0.805

Table S11 Most abundant putative plant pathogen ASVs that were present in at least 10% of experimental microcosms and were absent in unplanted control microcosms compared with experimental microcosms (i.e. were accumulated in the rhizosphere of the studied species). The table shows ASV taxonomy, the proportion of experimental microcosms where ASV was detected, and total sequence number. Genera associated with *Poaceae* plant hosts based on literature are highlighted in bold.

ASV ID	Genus	Species	Proportion of samples	Total abundance
ASV11	*Eocronartium*		0.78	76235
ASV269	***Pyrenophora***	***pseudoerythrospila***	0.44	2011
ASV492	*Fusarium*		0.18	787
ASV805	*Plectosphaerella*	*cucumerina*	0.39	348
ASV1126	***Ophiosphaerella***		0.19	196
ASV1310	*Mollisia*	*novobrunsvicensis*	0.13	151
ASV1416	*Ganoderma*	*australe*	0.23	132
ASV1752	*Alternaria*	*metachromatica*	0.14	94
ASV1784	***Ustilago***	***kummeri***	0.23	92
ASV1788	*Plectosphaerella*	*cucumerina*	0.13	92

Table S12 Effects of defoliation (Clipping) and genotypic diversity (Mix) of *Anthoxanthum odoratum* on fungal relative abundance estimated using linear mixed models. Separate models were fitted for all fungi as well as for functional subgroups. The genotypic composition of the pot was included as an intercept-level random factor, and the sequence depth as a covariate to account for potential differences in sequencing effort. Degrees of freedom are reported for fixed effects (Df) and model residuals (Df.res). F- and P- values are shown; P-values lower than 0.1 are shown in italic and lower than 0.05 in bold.

Response	Term	F	Df	Df.res	*P*
Pathogens	Clipping	**13.31**	**1**	**37.0**	**0.001**
	Mix	0.07	1	37.1	0.788
	log(seq_depth)	0.51	1	73.4	0.478
	Clipping*Mix	0.25	1	37.0	0.621
Pathogens without ASV11	Clipping	0.03	1	36.8	0.871
	Mix	0.02	1	37.4	0.900
	log(seq_depth)	**6.32**	**1**	**73.2**	**0.014**
	Clipping*Mix	*3.90*	*1*	*36.8*	*0.056*
AM fungi	Clipping	0.55	1	37.1	0.464
	Mix	0.22	1	37.1	0.644
	log(seq_depth)	0.11	1	72.4	0.737
	Clipping*Mix	<0.01	1	37.1	0.960
Saprotrophs	Clipping	**5.70**	**1**	**37.1**	**0.022**
	Mix	1.77	1	37.0	0.192
	log(seq_depth)	2.23	1	72.0	0.140
	Clipping*Mix	1.45	1	37.1	0.236
